# SARS-CoV-2 wastewater concentration and linked longitudinal seroprevalence: a spatial analysis of strain mutation, post-COVID-19 vaccination effect, and hospitalization burden forecasting

**DOI:** 10.1101/2023.01.06.23284260

**Published:** 2023-01-07

**Authors:** Rochelle H Holm, Grzegorz A Rempala, Boseung Choi, J Michael Brick, Alok R Amraotkar, Rachel J Keith, Eric C Rouchka, Julia H Chariker, Kenneth E Palmer, Ted Smith, Aruni Bhatnagar

**Affiliations:** Christina Lee Brown Envirome Institute, School of Medicine, University of Louisville, Louisville, KY 40202, USA; Division of Biostatistics, College of Public Health, The Ohio State University, Columbus, OH 43210, USA; Division of Big Data Science, Korea University, Sejong, South Korea; Biomedical Mathematics Group, Institute for Basic Science, Daejeon, South Korea; Westat, Inc., Rockville, MD 20850, USA; Christina Lee Brown Envirome Institute, School of Medicine, University of Louisville, Louisville, KY 40202, USA; Christina Lee Brown Envirome Institute, School of Medicine, University of Louisville, Louisville, KY 40202, USA; Department of Biochemistry and Molecular Genetics, University of Louisville, Louisville, KY 40292, USA; KY INBRE Bioinformatics Core, University of Louisville, Louisville, KY 40202, USA; Department of Biochemistry and Molecular Genetics, University of Louisville, Louisville, KY 40292, USA; KY INBRE Bioinformatics Core, University of Louisville, Louisville, KY 40202, USA; Center for Predictive Medicine for Biodefense and Emerging Infectious Diseases, University of Louisville, Louisville, KY 40202, USA; Department of Pharmacology and Toxicology, School of Medicine, University of Louisville, Louisville, KY 40202, USA; Christina Lee Brown Envirome Institute, School of Medicine, University of Louisville, Louisville, KY 40202, USA; Christina Lee Brown Envirome Institute, School of Medicine, University of Louisville, Louisville, KY 40202, USA

**Keywords:** COVID-19, environmental surveillance, epidemiology, sewer, vaccine, wastewater-based epidemiology

## Abstract

**Background:**

Since early in the COVID-19 pandemic, SARS-CoV-2 wastewater concentration has been measured as a surrogate for community prevalence. However, our knowledge remains limited regarding wastewater concentration and effects of the COVID-19 vaccination on overall disease burden as measured by hospitalization rates.

**Methods:**

We used weekly SARS-CoV-2 wastewater concentration with a stratified random sampling of seroprevalence, and spatially linked vaccination and hospitalization data, from April to August 2021. Our susceptible (*S*), vaccinated (*V*), variant-specific infected (I_1_ and I_2_), recovered (*R*), and seropositive (*T*) model (*SVI*_*2*_*RT*) tracked prevalence longitudinally. This was related to wastewater concentration for a spatial analysis of strain mutation, vaccination effect, and overall hospitalization burden.

**Findings:**

We found strong linear association between wastewater concentration and estimated community prevalence (r=0·916). Based on the corresponding regression model, the 64% county vaccination rate translated into about 57% decrease in SARS-CoV-2 incidence. During the study period, the estimated effect of SARS-CoV-2 Delta variant emergence was seen as an over 7-fold increase of infection counts, which corresponded to over 12-fold increase in wastewater concentration. Hospitalization burden and wastewater concentration had the strongest correlation (r=0·963) at 1 week lag time. We estimated the community vaccination campaign resulted in about 63% reduction in the number of daily admissions over the study period. This protective effect was counteracted by the emergence of SARS-CoV-2 Delta strain mutation.

**Interpretation:**

Wastewater samples can be used to estimate the effects of vaccination and hospitalization burden. Our study underscores the importance of continued environmental surveillance post-vaccine and provides a proof of concept for environmental epidemiology monitoring.

## 1. Introduction

There is an increasing realization that the current methods of disease monitoring based on individual testing may be insufficient to effectively combat the new, possibly much more infectious, severe acute respiratory syndrome coronavirus 2 (SARS-CoV-2) variants. This leaves public health researchers and policy makers in search for more reliable methods of measuring SARS-CoV-2 prevalence in communities and especially those not involving the (expensive) process of collecting individual level data. Wastewater concentration, when properly calibrated, can be a surrogate for the virus community prevalence analysis.^1,2^ Wastewater epidemiology promises an exciting opportunity to estimate community disease prevalence even with asymptomatic, vaccine preventable, disease.^2,3^ However, the handful of recent studies considering a relationship between SARS-CoV-2 wastewater concentration and the COVID-19 vaccine have relied almost exclusively on statistical models calibrated with publicly available COVID-19 clinical case data.^4–8^ These data run the risk of biased underrepresentation of asymptomatic individuals who may not seek testing, or individuals testing in settings where reporting is low or not required.^9^ In this study we consider this question in the context of randomized seroprevalence surveillance, combining mechanistic and statistical frameworks to obtain a more robust and realistic answer.

We used repeated cross-sectional community-wide stratified randomized sampling to measure SARS-CoV-2 nucleocapsid (N) specific IgG antibody-based seroprevalence in Jefferson County, Kentucky (USA), from April through August 2021 to determine post-vaccine community prevalence at a sub-population scale. We then related this to a statistical linear model and the available sub-population weekly wastewater surveillance data which thus yielded an explicit impact of vaccination and seroimmunity on SARS-CoV-2 wastewater concentration estimate, while controlling for prevalence in different epidemic phases. The latter may be easily translated into other important public health indicators such as the patterns of hospitalization.

## 2. Methods

### 2.1 Seroprevalence

Community-wide stratified randomized seroprevalence sampling was conducted in four waves from April to August 2021 in Jefferson County, Kentucky (USA) which is also the consolidated government for the city of Louisville. Seroprevalence sampling was both before and during vaccination, but this analysis only considers the period after COVID-19 vaccines were made widely available to the public (N=3436). An address-based sampling frame was used to build four geographic zones. Invitations (~30,000 per wave) were mailed to sampled households and one random adult was selected to join the study. Participants completed an online consent form and survey and scheduled an in-person appointment for testing at a mobile site. In some cases, due to the timing of sampling waves, respondents may have had only the first of a two-dose vaccine series. Owing to elevated levels of vaccinated respondents in our study (~90%), seroprevalence was measured by response to IgG N antibodies.^10^ It was assumed over the study period vaccination induced antibodies do not decay below detection. The nucleocapsid (N) IgG test sensitivity was 65% and the specificity was 85%. The seroprevalence sampling by geographical zones are described in more detail in the Supplemental Material section S1.

### 2.2 Wastewater SARS-CoV-2 concentration

Wastewater samples were collected twice per week from five wastewater treatment plants (N=520; Supplemental Material section S2) from April to August 2021. From an influent 24-hour composite sampler, 125 ml of subsample was collected and analyzed for SARS-CoV-2. In a few cases due to an equipment malfunction, a grab sample was collected. The geographic area and population serviced by a wastewater treatment plant comprises a sewershed, the zone for which we consider in our model analysis across a range of population sizes, income levels and racial and ethnic diversity.^2^ Analysis used polyethylene glycol (PEG) precipitation with quantification in triplicate by reverse transcription polymerase chain reaction (RT-qPCR).^11^ Data for SARS-CoV-2 (N1) are reported as weekly average copies/ml of wastewater with a threshold value for N1 assays of 7·5 copies/ml.

### 2.3 Administrative COVID-19 data

Administrative data on COVID-19 vaccination and infected individuals’ hospitalization was provided by the Jefferson County health authority, Louisville Metro Department of Public Health and Wellness (LMPHW), under a Data Transfer Agreement. Vaccination data were geocoded to the sewersheds using ArcGIS Pro version 2·8·0 (Redlands, CA). Daily hospitalization data was only available aggregated at a county level.

### 2.4 Analytical model

The hybrid model for estimating the effect of vaccination and strain mutation on longitudinal wastewater concentration was developed by combining a compartmental ecological model with a statistical linear model. The former was used to longitudinally estimate population prevalence from the observed cross-sectional rates of seropositivity. We assumed the overall vaccination pattern as reported by the county, with the overall adult vaccination rate reaching 64%^12^ by the end of the study period. The hybrid model was used to relate the ecological model prevalence to the wastewater data. The ecological model, *SVI*_*2*_*RT*, tracked longitudinally the proportions of individuals who were susceptible (*S*), vaccinated (*V*), infected with non-Delta (*I*_1_), and Delta variant (*I*_2_), recovered (*R*), or seropositive (*T*). The model is described in more detail in the Supplemental Material section S3. We note that a version of this model that did not account for vaccination or mutation was considered in our earlier work.^2^

#### 2.4.1 Regression model for wastewater concentration of SARS-CoV-2

Upon estimating the parameters in the model, we compared the model-calculated prevalence estimates for SARS-CoV-2 infections and vaccination levels with the wastewater concentration levels of SARS-CoV-2 (N1) normalized by pepper mild mottle virus (PMMoV).^13^ Bayesian linear regression was performed both on the county aggregated data and stratified by sub-community wastewater treatment plant zones (sewersheds). To improve the regression model stability, we used weekly average prevalence counts from the *SVI*_2_*RT* model as the explanatory variable, and weekly aggregated average wastewater concentrations as the single outcome variable. We assigned non-informative priors to all regression parameters. Specifically, the non-informative Cauchy distribution was assigned to regression coefficients, and the non-informative gamma prior was assigned to the dispersion parameter error term.

#### 2.4.2 Estimating vaccination, strain mutation and hospitalization effects

The strong statistical significance of the regression model relating prevalence and wastewater concentration allowed for indirect estimation of the effect of population vaccination and virus mutation. Under the assumption the relationship between the wastewater concentration and the prevalence is not confounded by the vaccination and mutation process, we used the original regression equation derived from the collected wastewater and seroprevalence data to estimate the wastewater concentrations over time. To estimate the vaccination effect, we compared these concentrations with hypothetical ones obtained when the vaccination term was zeroed out in the model. In a comparable manner, we estimated the effect of the introduction of the Delta-variant. Finally, we performed the longitudinal, regression-based analysis relating the community hospitalization to observed wastewater concentrations. In all three analysis we quantified the effects by calculating the size of the effects relative to the factual (observed) states.

#### 2.4.3 Competing risk model with two different virus strains

Wastewater samples were prepared for whole genome sequencing^11, 14^, and the proportion of observed SARS-CoV-2 variants was estimated for each sewershed based on the frequency of mutations specific to each variant.

Two variants were present in the study area during the study period: Alpha was dominant April to July, while Delta was dominant July and August.^11, 14^ To reflect the infections before and after the emergence of the Delta variant, we incorporated into our *SVI*_2_*RT* model two different infection compartments (*I*_1_ and *I*_2_) reflecting the infection competition between two different strains of the virus.

### 2.5 Ethics

For the seroprevalence and data provided by the LMPHW under a Data Transfer Agreement, the University of Louisville Institutional Review Board approved this as Human Subjects Research (IRB number: 20·0393). For the wastewater data, the University of Louisville Institutional Review Board classified this as non-human subjects research (reference #: 717950).

## 3. Results

### 3.1 Wastewater regression

The results of Bayesian regression analysis relating the prevalence estimated from the model, and the observed wastewater levels, both in aggregation and by sewershed area, show a significant trend that is well summarized by the corresponding posterior regression line. Normalized SARS-CoV-2 by PMMoV provided more reliable and stable longitudinal concentration readings than using SARS-CoV-2 N1 concentration alone. For the aggregate model, the estimated intercept is 5·563 × 10^−4^ (CI =(−9·903 × 10^−4^, −1·250 × 10^−4^)) and the slope is 0·453 (CI=(0·374, 0·529)). Overall, we see the regression model fits well with *R*^2^=0·909. See Supplemental Material Figure S3.2 and S3.3.

### 3.2 Effect of vaccination on disease incidence and wastewater concentration

**Figure 1.**
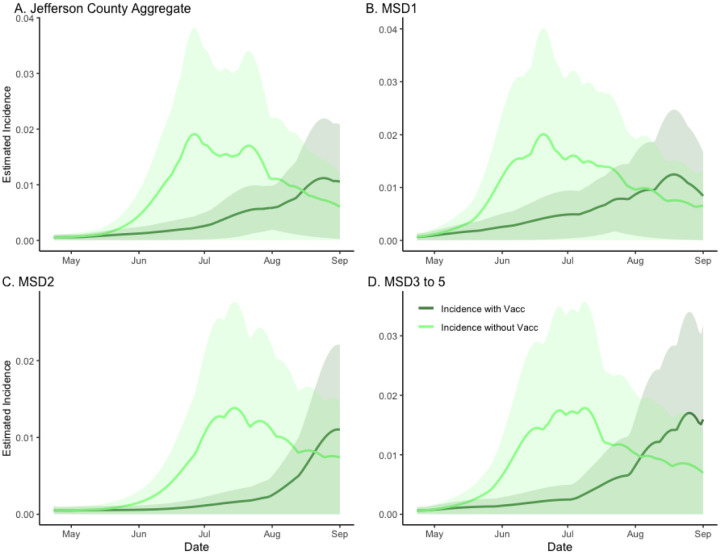
The estimated effect of vaccination on incidence in sewersheds of Jefferson County, KY (USA). The dark green line is the factual *SVI*_2_*RT* model estimated incidence (with vaccination), and the light green line is the corresponding counter-factual estimated incidence with vaccination effect zeroed out. The shaded areas represent 95% credible intervals. The panels compare the vaccination effect in Jefferson County (Panel A) as well as stratified by sewershed (Panels B–D).

We first compared the estimated incidence of the model under two different vaccination scenarios (observed 64% vaccination rate and counter-factual 0% vaccination rate) while adjusting for the Delta variant emergence (Figure 1). The peak and the overall temporal dynamics are different under the two scenarios across each location. To better quantify these differences, we calculated the location-specific vaccination effects as the ratios of the areas under two scenario curves (with-vaccination area over without-vaccination area). The value obtained for the aggregated data was 0·429 (CI= (0·405, 1)), with the remaining sewershed specific effects being even stronger at (Figure 1; panels B–D) 0·532 (CI= (0·515, 1)), 0·367 (CI= (0·366, 0·785)), and 0·555 (CI= 0·555, 1)), respectively. Based on converting these ratios to excess incidence, we conclude that without vaccination, we would expect to see the incidence increase of about 133% above the observed level in Jefferson County (panel A) and about 88%, 172%, and 80% in respective sewershed areas (Figure 1; panels B–D, see also S3).

**Figure 2.**
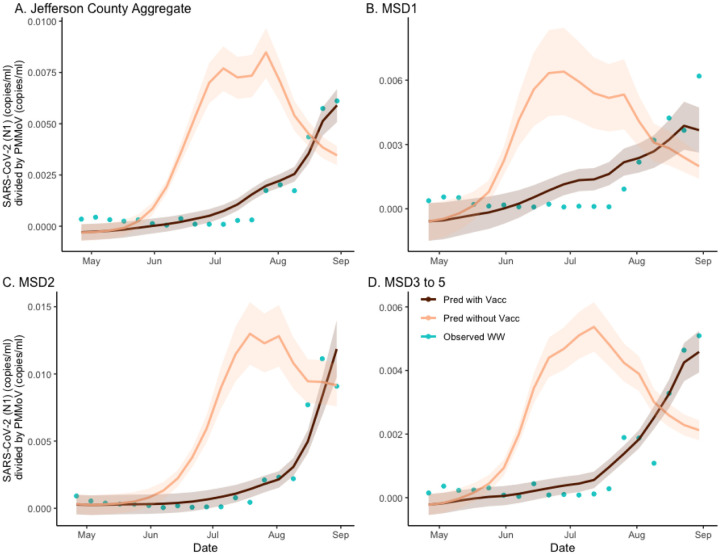
The estimated effect of vaccination on SARS-CoV-2 wastewater concentration normalized by pepper mild mottle virus in sewersheds of Jefferson County, KY (USA). The deep brown line is the regression-based fit to the wastewater concentration data and the light brown line is the prediction of wastewater concentration using synthetic prevalence from model with vaccination effect zeroed out. The shaded areas represent 95% credible intervals. The blue dots are observed weekly average wastewater concentrations The panels compare the vaccination effect on wastewater concentration for Jefferson County (Panel A) as well as stratified by sewershed (Panels B–D).

To obtain estimates of the vaccination effects on the wastewater concentrations, we developed a hybrid inferential model combining the wastewater regression (see Sec 3.1) equation with the *SVI*_2_*RT* estimated prevalence, under two different vaccination scenarios (factual 64% rate and counter-factual 0% rate) (Figure 2). Note that the usage of *SVI*_2_*RT* (which accounts for the effect of different virulence of the two different SARS-CoV-2 strains) automatically adjusted our analysis for the Delta variant emergence. As the estimated prevalence from the model and the normalized wastewater concentration are highly correlated (see Sec 3.1), the hybrid model is seen to fit data well. As before, to quantify the location-specific vaccination effects, we calculated the location-specific ratios under two curves in an analogous way as when quantifying the vaccination effect on the disease incidence. The ratios of the areas under the two curves, under factual (vaccinated) and counterfactual (unvaccinated) scenarios, were computed. The Jefferson County (Figure 2; panel A) ratio was equal to 0·358 (CI= (0·333, 0·381)), and the remaining sewershed area ratios (Figure 2; panels B–D) were equal to, respectively, 0·457 (CI= (0·388, 0·537)), 0·276 (CI= (0·260, 0·296)), and 0·426 (CI= 0·407, 0·446)). The estimate of excess wastewater virus without vaccination is estimated as 179%, 119%, 262%, and 135%, respectively (Supplemental material section S3).

### 3.3 Effects of virus mutation on disease incidence and wastewater concentration

The time periods during which the Alpha and Delta variants were dominant in each sewershed are are shown in Table S4.1. The onset of Alpha as the dominant variant simultaneously occurred at each site on 3/30/21 and lasted a variable number of weeks before dying out and eventually becoming replaced by Delta. However, Delta had a more gradual introduction into the sewersheds, beginning as the dominant variant on 7/12/21 in two of the sites and not showing up as the dominant variant in one of the sites until two weeks later. Our gradual linear switch from Alpha to Delta is similar to observations in other wastewater data, including a study of 94 sites within Austria.^22^ Interestingly, Delta’s dominance as the major variant ended simultaneously on 8/30/21 in all five sites, prior to the later emergence of Omicron in the sewersheds in December 2021. More recently, we have reported on the re-emergence of Delta in the MSD03 site specifically during the Omicron wave,^14^ which indicates the persistence of specific variants in wastewater can be variable in nature, and are likely influenced by a number of factors, including incidence and vaccination rates.

**Figure 3.**
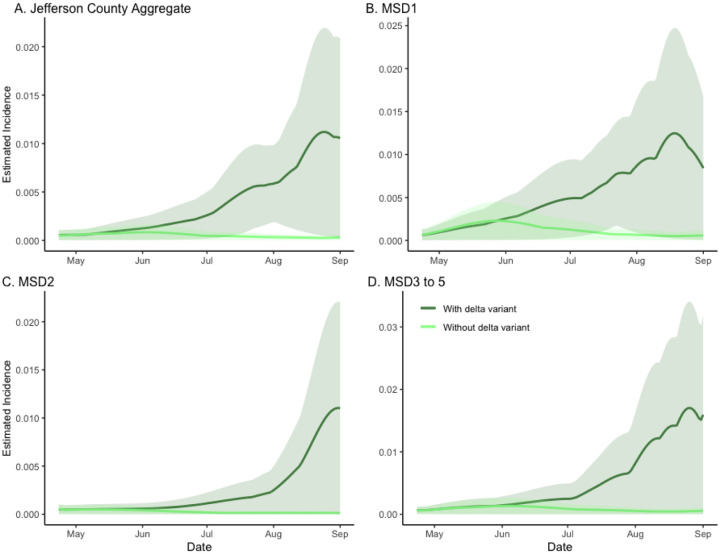
The model-based analysis of the Delta-variant effect on SARS-CoV-2 incidence rate estimates in sewersheds of Jefferson County, KY (USA). The dark green line is the estimated factual full model incidence (both Alpha and Delta variants present), and the light green line is the counterfactual incidence estimated from the model with no Delta variant. The shaded areas represent 95% credible intervals. The panels compare the vaccination effect in Jefferson County (Panel A) as well as stratified by sewershed (Panels B–D).

In the analysis, we assumed a 20% higher infectivity of the SARS-CoV-2 Delta variant as compared to its Alpha predecessor.^15^ In the counterfactual model (light curve), where only the Alpha variant was present, the epidemic was seen to dissipate, indicating the basic reproduction number less than one. This was in stark contrast with the factual, full model fit (with both Alpha- and Delta- variants present), where the incidence (dark curve) was seen to rise rapidly (Figure 3). As in the previous section, to quantify the difference between the two curves, which we interpret as measuring the effect of introducing the Delta mutation, we calculated the ratio of areas under the two curves in each panel, obtaining the values of 7·32 (CI = (7·05, 20·13)), 4·40 (CI = (4·33, 7·64)), 8·58 (CI = (1, 8·60)), and 6·15 (CI = (1, 6·16)) for the aggregate, MSD1, MSD2, and MSD3–5 regions respectively (corresponding to panels A–D). The estimate of the decrease in total incidence without mutation is found as 86%, 77%, 88%, and 84%, respectively.

**Figure 4.**
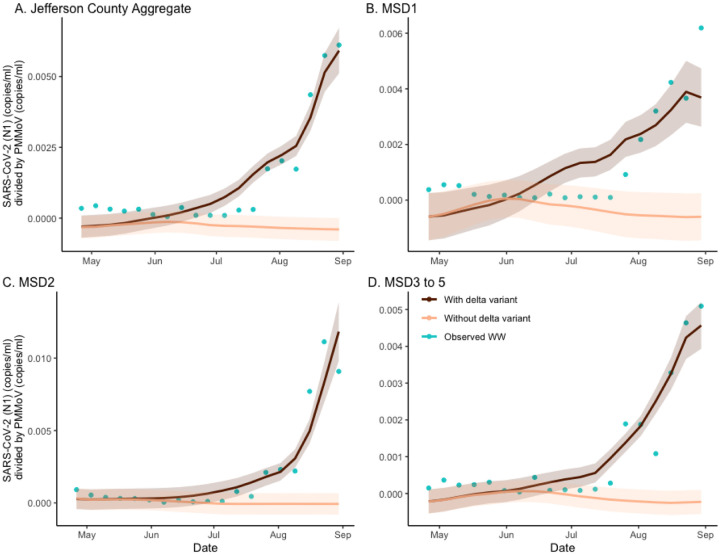
The estimated effect of Delta variant on SARS-CoV-2 wastewater concentration normalized by pepper mild mottle virus in sewersheds of Jefferson County, KY (USA). The deep brown line is the regression-based fit to the wastewater concentration and the light brown line is the prediction of wastewater concentration using synthetic prevalence from the model with the Delta variant effect zeroed out. The shaded areas represent 95% credible intervals. The blue dots are observed weekly average wastewater concentration. The panels compare the mutation effect on wastewater concentration for Jefferson County (Panel A) as well as stratified by sewershed (Panels B–D).

To identify the effect of the Delta variant emergence on the observed wastewater concentration, we again applied the hybrid model discussed in the previous section. In the current analysis, the regression model was applied to predict the longitudinal wastewater concentrations from both factual (both variants present) and counterfactual prevalence data (no Delta variant). The results are depicted in the panels of Figure 4 both for the aggregated and sewershed-specific analysis. As with the analysis of the vaccination effects, here we also considered the ratios of areas under the corresponding curves as measures of Delta variant effects in specific locations. Based on the location-specific ratio values of 12·569 (CI = (11·487, 13·914)), 6·235 (CI = (5·290, 7·891)), 14·932 (13·351, 16·898), and 8·413 (CI = (7·654, 9·351)), corresponding to aggregated and sewershed-specific curves, the estimate of excess wastewater virus due to Delta mutation is founded as 92%, 84%, 93%, and 88% respectively. Further analysis is provided in Supplemental Material Table S3.3.

### 3.4 Forecasting hospitalization rates based on wastewater concentrations

**Figure 5.**
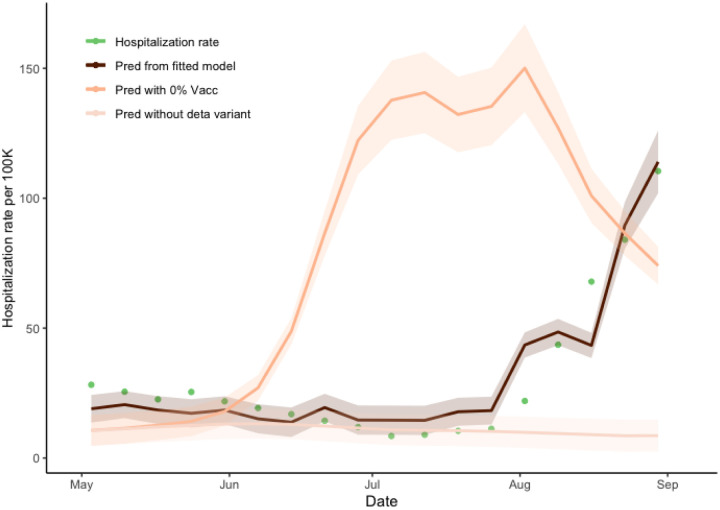
Time lag-dependent analysis of the relationship between hospitalization rate and wastewater concentration, Jefferson County, KY (USA). Predictions and 95% confidence intervals of hospitalization rate regressed on week-lagged variables of the weekly average of wastewater concentration according to the changes of the vaccination proportion of the community. The dark line represents the prediction using the observed wastewater concentrations with 64% of community vaccination. The lighter line represents the prediction using the wastewater concentrations obtained from the model under zero community vaccination. The lightest line represents the prediction under the counterfactual modified model with the Delta-infected model compartment zeroed-out (no Delta variant present). The green dots represent the weekly average of the observed hospitalization rate. The ratios of the areas between the prediction from the fitted model and of no vaccination are 0·368, and in the absence of the Delta variant is 2·63, respectively.

Hospitalization estimates under both vaccinated (64% vaccination rate) and unvaccinated (0% vaccination rate) scenarios were obtained by applying a hierarchical regression model where we first regressed wastewater concentration on the model prevalence and then regressed hospitalization counts on the wastewater concentrations (Figure 5). As hospitalization is likely to occur sometime after symptom onset, we used the 1-week lagged-regression model where the length of the lag was based on the overall model fit criteria. The fitted intercept and slope coefficients were 1·284 × 10^−4^ (std=2·279 × 10^−5^) and 0·176 (std=0·0119) for vaccinated and unvaccinated scenarios respectively, with the R-square of 0·928. The maximal number of the observed daily average hospitalizations under vaccination scenario was 110·4 per weekly average (actual 122·0 in daily) at the end of August. However, without vaccination, the maximum predicted number of weekly average hospitalizations increased to 150·3. The ratios between the areas under the prediction curves with and without vaccination were 0·368 (CI = (0·413, 0·458)), indicating a 170% increase in the number of hospitalizations when no vaccine would be present. In a comparable way, we obtained the hospitalization estimate without the Delta variant mutation. The ratio of the areas under the two graphs (with and without the Delta variant mutation) is 2·632 (CI = (2·382, 5·573)), indicating a 62% decreasing in the hospitalization rate.

## Discussion

The results of our large study (N=3436) have shown the importance of environmental surveillance post-vaccine in urban and sub-urban areas; removing bias of publicly available clinical case data by using antibody positivity with four waves of sequential community-wide stratified randomized sampling data. Despite our focus on the Jefferson County example, it should be emphasized the model described here is readily applicable to other locations worldwide. Although our model was run with both the N1 analysis and adjusted N1 analysis, we learned the model provided reduced uncertainty with adjustment. Indeed, the vaccination effect estimates bring the related issue of refined localized model application such as high levels of tourism that may affect community vaccination levels and related observed wastewater concentrations.^8^ Here we have presented real world evidence that, in fact, wastewater surveillance may be also used to estimate both the effects of a community intervention, like the vaccination campaign and the effects of disease evolution, and emergence of a new viral strain mutation.

The COVID-19 vaccine has been highly effective.^16, 17^ There is a need for increased reliance on wastewater as a proxy for community disease impact being built from actual community level data over time for other vaccine preventable diseases affecting human health. When 90% of the student population of a college campus was vaccinated, SARS-CoV-2 in wastewater decreased;^4^ but that building level generalization was not replicated in our community-wide survey over a longer period. In contrast to our findings, Nourbakhsh et al.^18^ found dissimilar trajectories from clinical and wastewater data ratios once vaccination was introduced. We suspect this difference is explained by the bias of relying on clinical data and home testing kits which became more widely available for the studied period when compared to earlier in the pandemic and with no requirement, or in some cases option, for reporting. Whereas the Nourbakhsh et al.^18^ study included only publicly reported case data, the randomized selection of community participants in our study population were a comparatively less biased data source for this post-vaccine period.

Our model compares to the work of Jiang et al.^19^ in that our analysis also provided estimates of prevalence, however our estimates are based on a statistical random sample (not a clinical sample) and our regression model has a simple and explicit formula relating prevalence to observed wastewater concentrations. Our model further confirms the findings in Hegazy et al.^6^ implying the Delta variant emergence has strengthened the relationship between wastewater and hospitalization rates. Our analysis provides a further proof of concept that our wastewater regression model could be used (after proper calibration) with other similar data to provide surrogate measures of SARS-CoV-2 prevalence in the community without the necessity for individual testing. The regression prediction correlates well with the estimated prevalence with a correlation coefficient of 0·916 (CI = (0·764, 0·976). The hospital burden findings of Wang et al.^20^ also compared well to our work; our results showed access to a voluntary community vaccine that reached a coverage level of 64% of the adult population decreased community hospitalizations by approximately 170%.

Yaniv et. al^5^ described the emergence of peaks of positivity rates, showing they corresponded to introduction of new variants. In addition, they noted how vaccination rates and a second booster helped to control Alpha, while an increase in a third booster was found to lead to a decline in Delta. When vaccination levels increase to higher coverage, overall reported incidence may decline, even though the levels in wastewater remain high.^7^ This leads to the hypothesis that circulation among vaccinated individuals creates a level of selective pressure making variants with transmission and vaccine escape more advantageous. In our wastewater data, we find in periods of decline for a specific variant we see more diversity in the overall mutations, indicating both a selection against the variant in decline (typically due to vaccinations and boosters), and a similar advantageous selection for emerging variants.

Our study used five sub-community scales based on the existing wastewater infrastructure allowing observation of regional trends but also the aggregation of data for a countywide picture. We found the antibody positivity varied by the sewershed. The antibody-positive individuals were lowest in sewershed MSD1 and highest in sewershed MSD3–5 (10·0% for aggregate, 8·6% for MSD1, 9·2% for MSD2, and 12·0% for MSD3–5), indicating previous infection may have been higher in the rural portions of the county compared to the urban core. There are many factors differentiating these sewershed areas that could have produced these differences. These include population sizes and demographics, or presence of stormwater or industrial discharge being combined with household sewer water. These differences between MSD1 to 5 provide evidence of the benefit of observing results at a sub-county level, rather than only considering MSD1 representing the urban core and the largest sewershed zone.

The trajectory of the pandemic and public health response would benefit from new methods less dependent on continuous individual clinical testing. For replication of this SARS-CoV-2 model, wastewater sampling, stratified random sampling of seroprevalence, and spatially linked vaccination data are required; the model is flexible enough to allow additional variant-specific variables. The promise of this model is if we just have wastewater concentration, we can we predict the effect of vaccination and allow fine-tuned, and milestone driven, public health response. Our model also provides a further positive response for public health offices of the significant role of the vaccine, our competition model shows the epidemic would have been bigger and earlier without vaccine access.

## 4. Limitations

Our study has several limitations. The proportion of vaccinated respondents was larger than the greater community (~90% vs. 64%) reflecting that even a probability-based sample relies on volunteer participants and vaccinated individuals may also have different health-research behaviors. Vaccine information was self-reported. Natural infection of a combined vaccinated and unvaccinated population (and in the absence of another way to verify vaccination) was based on antibody titers of IgG N, an assay that has 65% sensitivity and 85% specificity, with inevitable under-estimation of infection prevalence. While our serosurvey only captured adults, wastewater testing included minors. COVID-19 infected individuals can in rare instances shed fecal SARS-CoV-2 RNA up to 7 months post diagnosis;^21^ viral shedding of each SARS-CoV-2 variant in relation to days after vaccination, or whether a primary or booster series had been taken, is not well defined as the vaccination series guidance extends during the pandemic and thus was not included in our model.

## 5. Conclusion

Our work indicates it is possible in certain conditions to use wastewater-based epidemiology to assess both the immunity acquisition in the community due to natural recovery and vaccination as well as the effect of new variants emergence and associated immune evasion to the currently available COVID-19 vaccines. The effects of vaccination on wastewater concentration as well as on community incidence of SARS-CoV-2 was substantial in Jefferson County. According to our analysis, without vaccination one would expect about 133% of excess infections over the period of study, which corresponds to a 179% of excess wastewater concentration. The effect of Delta mutation was similarly substantial. We estimated, over the study period in Jefferson County, without Delta mutation the amount of overall infection would decrease on average by 86% which corresponds to a 92% decrease in wastewater N1 normalized by PMMoV concentration. The correspondence between wastewater concentration and the number of hospitalizations was found to be strongest with the time lag for about 7 days and correlation = 0·963. Based on the regression model we estimated the effects of vaccination and mutation on hospitalization rate. According to the model, without vaccination one would expect about 171% increase and without mutation about 62% decrease in hospitalization rate. Using the fitted regression model for hospitalization, the predictions of hospitalization rates are at 50, 100, and 150 per 100K when the normalized wastewater concentrations are 0·0021, 0·0050, and 0·0078 N1 normalized by PMMoV, respectively. Our large, randomized, serosurvey suggests using the mechanistic, population level, vaccination model (*SVI*_2_*RT*) coupled with longitudinal wastewater sampling estimated the effect of vaccination on the prevalence rate in the community over the period of several months during the second and third wave of COVID-19 pandemic, in the absence of clinical data. The model can also be used to estimate the effects of vaccination and new variants emergence on the hospitalization rate and on peak hospital beds utilization.

## Supplementary Material

1

## Data Availability

The seroprevalence, wastewater concentration, and hospitalization information data used in the study can be accessed from the website https://github.com/cbskust/DSA_Seroprevalence. The computer code that implemented our model-based analysis will be made available immediately after publication.

## Research in context

### Evidence before this study:

We searched Web of Science and PubMed for all available articles until August 24, 2022, using the search terms [“seroprevalence” or “antibody”] AND [“wastewater”] AND [“vaccination”]. We examined only English literature. We identified 59 studies. None of these studies considered community level randomized antibody testing paired with vaccination and SARS-CoV-2 wastewater concentration data. Where wastewater and vaccination status have been historically linked is with *Poliomyelitis*, in the known spatial scale of vaccination rates and using wastewater surveillance to confirm presence/absence of community infection. Few studies considered hepatitis A antibodies in workers exposed to sewage to guide vaccination campaigns. We also found some non-human subject research. To our knowledge, there is no real-world setting SARS-CoV-2 study where quantified wastewater concentration is linked to a large longitudinal stratified randomized seroprevalence sampling at a sub-population scale and the population has voluntary access to a vaccination reducing hospitalization burden.

### Added value of this study:

To our knowledge, this study provides the first analysis of SARS-CoV-2 wastewater concentration as the basis for estimating subpopulation vaccination and virus mutation effects, and hospitalization burden in any country. We used actual seroprevalence data from a large US urban area that was rigorously collected through statistical sampling, to obtain a longitudinal estimate of disease prevalence. We then used a statistical model relating prevalence to wastewater concentration for a spatial analysis of vaccination, virus mutation effects, and for forecasting hospitalization burden. The methodology developed in the current paper has a potential to improve both the effectiveness of monitoring and the predictive accuracy of wastewater-based surveillance systems.

### Implications of all the available evidence:

Our results show the potential of sustained environmental surveillance post-vaccine in urban areas and on removing bias in population-level estimates of prevalence of SARS-CoV-2 due to over-reliance on reported clinical testing data. The methodology presented here provides further proof wastewater monitoring can be successfully used as a tool for estimating both the community impact of changing disease patterns and various interventions over time. These findings have implications beyond current SARS-CoV-2 pandemic since our proposed approach is quite general and can be applied to other vaccine preventable diseases affecting human health in the absence of clinical testing data.
