# Supplementary material for: Wastewater and seroprevalence for pandemic preparedness: variant analysis, vaccination effect, and hospitalization forecasting for SARS-CoV-2, in Jefferson County, Kentucky
Source: medRxiv. 2023 Nov 28:2023.01.06.23284260. Originally published 2023 Jan 7. Preprint. [Version 2] doi: 10.1101/2023.01.06.23284260 (PMC9844017; doi:10.1101/2023.01.06.23284260)
Supplement: Supplement 1 [file NIHPP2023.01.06.23284260v2-supplement-1.pdf]

medRxiv preprint doi: https://doi.org/10.1101/2023.01.06.23284260; this version posted November 28, 2023. The copyright holder for this preprint (which was not certified by peer review) is the author/funder, who has granted medRxiv a license to display the preprint in perpetuity.

It is made available under a CC-BY 4.0 International license .

Supplementary information

Wastewater and seroprevalence for pandemic preparedness: variant analysis, vaccination effect, and hospitalization forecasting for SARS-CoV-2, in Jefferson County, Kentucky

Table of Contents

Appendix A. SARS-CoV-2 seroprevalence by wave and sewershed, Jefferson County, KY (USA)........ 28

Appendix B. Studied wastewater treatment plant zones (sewersheds), Jefferson County, KY (USA)........ 29

Appendix C. Population vaccination model (SVI_2RT)........ 32

Appendix D. Wastewater variant detection........ 56

medRxiv preprint doi: https://doi.org/10.1101/2023.01.06.23284260; this version posted November 28, 2023. The copyright holder for this preprint (which was not certified by peer review) is the author/funder, who has granted medRxiv a license to display the preprint in perpetuity.

It is made available under a CC-BY 4.0 International license .

Appendix A. SARS-CoV-2 seroprevalence by wave and sewershed, Jefferson County, KY (USA).

Table S1. **SARS-CoV-2 seroprevalence by wave and sewershed, Jefferson County, KY (USA).**

	Number of unvaccinated participants	Number of vaccinated participants	Number of participants positive for SARS-CoV-2 nucleocapsid (N1) specific IgG antibodies	Estimated posterior average seroprevalence per 10^5 people (95% credible interval)	Estimated posterior average prevalence per 10^5people (95 % credible interval)
Overall					
MSD1	98	1464	132	9153 (3772, 14533)	533 (38, 1028)
MSD2	134	800	81	5427 (1848, 9006)	336 (0, 672)
MSD3–5	86	721	83	8410 (2016, 14804)	588 (0, 1177)
Total	318	2985	296	7596 (2639, 12554)*	465 (7, 923)*
Wave A					
MSD1	27	372	24	2249 (1686, 2811)	62 (1, 122)
MSD2	31	208	25	2277 (1751, 2803)	49 (0, 97)
MSD3–5	13	113	19	2284 (1682, 2887)	53 (1, 106)
Total	71	713	68	2265 (1710, 2820)*	55 (0, 110)*
Wave B					
MSD1	26	370	17	2927 (2042, 3813)	197 (7, 386)
MSD2	40	192	17	2611 (1832, 3391)	74 (0, 149)
MSD3–5	23	170	8	2952 (1816, 4089)	271 (0, 542)
Total	89	733	42	2809 (1917, 3701)*	161 (0, 334)*
Wave C					
MSD1	16	309	22	6261 (2537, 9984)	618 (43, 1193)
MSD2	29	179	11	3696 (1860, 5531)	197 (0, 394)
MSD3–5	15	171	13	7565 (2048, 13083)	456 (0, 911)
Total	60	659	46	5473 (1952, 8994)*	430 (0, 875)*
Wave D					
MSD1	29	413	69	19572 (8999, 30145)	810 (4, 1617)
MSD2	34	220	28	12408 (1872, 22943)	834 (0, 1668)
MSD3–5	35	247	43	14746 (2188, 27304)	1440 (0, 2880)
Total	98	880	140	16048 (5155, 26941)*	918 (0, 1861)*

*Weighed average according to the population sizes of each sewershed zone.

medRxiv preprint doi: https://doi.org/10.1101/2023.01.06.23284260; this version posted November 28, 2023. The copyright holder for this preprint (which was not certified by peer review) is the author/funder, who has granted medRxiv a license to display the preprint in perpetuity.

It is made available under a CC-BY 4.0 International license .

Appendix B. Studied wastewater treatment plant zones (sewersheds), Jefferson County, KY (USA).

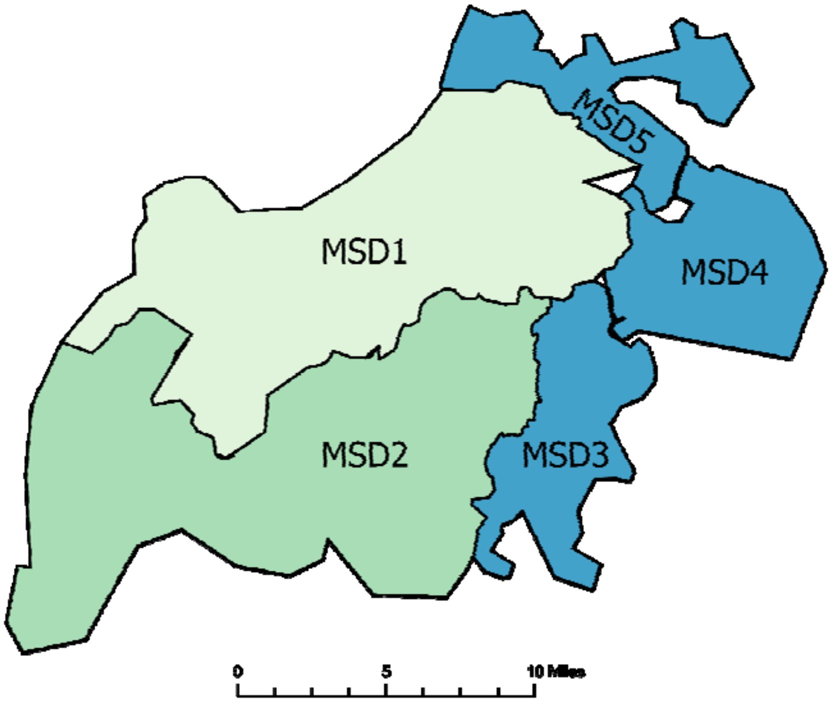

Figure S1. **Studied wastewater treatment plant sewersheds, Jefferson County, Kentucky (USA).**

medRxiv preprint doi: https://doi.org/10.1101/2023.01.06.23284260; this version posted November 28, 2023. The copyright holder for this preprint (which was not certified by peer review) is the author/funder, who has granted medRxiv a license to display the preprint in perpetuity.
It is made available under a CC-BY 4.0 International license .

Table S2. **Characteristics of studied wastewater treatment plant sewersheds of Jefferson County, KY (USA).**

**Sewershed**	**Income (USD$)**[a]	**Population**[a]	**Area (km^2)**	**Combined sewer**[b]
MSD1<br>Morris Forman Water Quality Treatment Center	54,138	349,850	280	Yes
MSD2<br>Derek R. Guthrie Water Quality Treatment Center	53,577	295,910	332	No
MSD3<br>Cedar Creek Water Quality Treatment Center	76,606	55,928	80	No
MSD4<br>Floyds Fork Water Quality Treatment Center	113,699	32,460	88	No
MSD5<br>Hite Creek Water Quality Treatment Center	106,769	31,269	67	No

[a]Based on 2018 U.S Census Bureau American Community Survey (ACS) block group data aggregated to the wastewater catchment areas with overlapping block group centroids. Income is mean median household. For more on wastewater site selection see Yeager et al., 2021.
[b]Combined sewers include wastewater and stormwater and SARS-CoV-2 concentrations may be expected to fluctuate more as a result.

medRxiv preprint doi: https://doi.org/10.1101/2023.01.06.23284260; this version posted November 28, 2023. The copyright holder for this preprint (which was not certified by peer review) is the author/funder, who has granted medRxiv a license to display the preprint in perpetuity.

It is made available under a CC-BY 4.0 International license .

References

1 Yeager R, et al. Wastewater sample site selection to estimate geographically resolved community prevalence of COVID□19: A sampling protocol perspective. *GeoHealth* **5**(7), e2021GH000420 (2021). https://doi.org/10.1029/2021GH000420

medRxiv preprint doi: https://doi.org/10.1101/2023.01.06.23284260; this version posted November 28, 2023. The copyright holder for this preprint (which was not certified by peer review) is the author/funder, who has granted medRxiv a license to display the preprint in perpetuity.

It is made available under a CC-BY 4.0 International license .

Appendix C. Population vaccination model (SVI_2RT)

The equation shown in (1) describes the time-evolution of the proportions of individuals who are susceptible (S), vaccinated (V), infected with Alpha variant (I_1), infected with Delta variant (I_2) removed (R), and seropositive (T). We assume the total initial population of susceptibles is large with a small initial fraction of infected. The model equations are:

$$\begin{aligned} \dot{S}_t &= -\beta S_t I_t^{(1)} - \beta^* S_t I_t^{(2)} - \alpha_t S_t, \\ \dot{V}_t &= \alpha_t S_t - \tilde{\beta} I_t^{(1)} V_t - \tilde{\beta}^* I_t^{(2)} V_t \\ \dot{I}_t^{(1)} &= \beta S_t I_t^{(1)} + \tilde{\beta} I_t^{(1)} V_t - \gamma I_t^{(1)}, \\ \dot{I}_t^{(2)} &= \beta^* S_t I_t^{(2)} + \tilde{\beta}^* I_t^{(2)} V_t - \gamma I_t^{(2)} \\ \dot{R}_t &= \gamma I_t^{(1)} + \gamma I_t^{(2)} - \delta R_t, \\ \dot{T}_t &= \delta R_t, \end{aligned} \qquad (1)$$

with the initial condition $S_0 = 1 - \rho(1.001) - \epsilon - \psi > 0, V_0 = 0$, $I_0^{(1)} = \rho > 0$, $I_0^{(2)} = \rho/1000$, $R_0 = \epsilon > 0$, and $T_0 = \psi > 0$.

Here, β and $\tilde{\beta}$ are the rates of infection of respectively, unvaccinated and vaccinated, and β^* and $\tilde{\beta}^*$ are the rates of infection according to Delta variant. As our compartment model has two infection compartments, it is called the variant competition model.[1] The observed data in this analysis do not have any information about infection from the Delta variant, and an increase in the number of parameters makes model estimation difficult and may lead to identifiability problems. So, we set β^* and $\tilde{\beta}^*$ at the values 50% higher than β and $\tilde{\beta}$.[2] The function of α_t represents a changing rate of vaccination over time. The vaccination process may be changed according to a policy or vaccine supply, so we set the vaccination rate α_t to match the empirical percentage of the vaccinated population in Jefferson County at the end of August 2021. Additionally, γ is the rate of recovery, and δ is the rate at which antibodies build to a detectable level after recovery. The SVI_2RT model parameters to be estimated are given by the vector $\theta = (\beta, \tilde{\beta}, \gamma, \delta, \rho, \epsilon, \psi)$.

To obtain the serial estimates of incidence and prevalence from the observed seropositivity levels in four waves of testing, we adapt the idea of an ODE-based survival model proposed recently.[3,4] According to that model, the scaled quantities $S_t, V_t, I_t^{(1)}, I_t^{(2)}, R_t, T_t$ may be considered as respective probabilities of a randomly selected individual in a large population, being either susceptible, vaccinated, infected with different virus variant, recovered, or seroprevalent at time t. Consequently, we consider the results $Z(t)$ of all individual antibody-based tests conducted at times t as independent Bernoulli variables:

$$Z(t) \sim \text{Ber}(T_t^*),$$

where $T_t^* = sensT_t + (1 - spe)(1 - T_t)$ is the specificity adjusted probability of a positive test. For our analysis, both $sens$ and spe are additional parameters to be estimated. We assigned the informative priors to $sens$ and spe from available clinical data.

Assuming at time t, n_t individuals are tested with k_t having positive results, the corresponding log-likelihood function is:

medRxiv preprint doi: https://doi.org/10.1101/2023.01.06.23284260; this version posted November 28, 2023. The copyright holder for this preprint (which was not certified by peer review) is the author/funder, who has granted medRxiv a license to display the preprint in perpetuity.

It is made available under a CC-BY 4.0 International license .

$\ell_t(\Theta) \propto k_t \log(T_t) + (n_t - k_t)\log(1 - T_t)$, (2)

where $\Theta = (\beta, \tilde{\beta}, \gamma, \delta, \rho, \epsilon, \psi, spe, sens)$ is the vector of parameters to be identified.
Given the testing data at $m \geq 1$ time points $t_1, \dots, t_m$, we then aim to find parameter values θ that maximizes the posterior log-likelihood function:

$\tilde{\ell}(\Theta) \propto \sum_{i=1}^{m} \ell_{t_i}(\Theta) + \log p(\Theta)$, (3)

where $p(\Theta)$ is the prior distribution on Θ to be determined from our previous work[3,4]. Hence, we seek the values of Θ that maximize our posterior log-likelihood function (3). The entire system (1) must be solved for each parameter combination.

S3.1 Incidence, prevalence, and seroprevalence estimation

Posterior serial estimates of the relative rates of incidence, prevalence, and seropositivity were obtained from the SVI_2RT model as the time-dependent vector:

$\text{Pred}_t = (-\dot{S}_t, V_t, I_t^{(1)}, I_t^{(2)}, T_t)$. (4)

Here $(S_t, V_t, I_t^{(1)}, I_t^{(2)}, T_t)$ is the family of trajectories of (1) evaluated at the posterior distribution of the vector Θ. In practice, the distribution of Pred_t is approximated by taking a random sample of size m from the converged MCMC sampler. In our case $m = 2000$. To obtain daily incidence rates (Inc_d) we have used the approximation $\dot{S}_t \approx S_{t+1} - S_t$ and consequently took $\text{Inc}_d = S_d - S_{d+1}$ where d corresponds to a specific day of interest. The estimated prediction counts were obtained by multiplying the rates in Pred_t by the appropriate population numbers.

medRxiv preprint doi: https://doi.org/10.1101/2023.01.06.23284260; this version posted November 28, 2023. The copyright holder for this preprint (which was not certified by peer review) is the author/funder, who has granted medRxiv a license to display the preprint in perpetuity.

It is made available under a CC-BY 4.0 International license .

Table S3. **Posterior mean estimates of the SVI_2RT model parameters in sewersheds of Jefferson County, KY (USA).** The area-specific Hamiltonian Markov chain Monte Carlo (MCMC) posterior estimates are based on seropositivity data aggregated across Jefferson County and stratified by sewersheds. The corresponding 95% credible bounds are provided in parenthesis. The results are based on MCMC implemented via *Rstan* library, with a 6000- and 2000-step burn-in.

	**Jefferson County Aggregated**	**MSD1**	**MSD2**	**MSD3–5**
$\boldsymbol{\beta}$	0.384 (0.301, 0.449)	0.375 (0.282, 0.443)	0.314 (0.234, 0.374)	0.353 (0.259, 0.427)
$\boldsymbol{\alpha}$	8.813×10^{-3} (64%)	0.010 (67%)	6.600×10^{-3} (55%)	0.013 (76%)
$\tilde{\boldsymbol{\beta}}$	0.317 (0.235, 0.381)	0.322 (0.238, 0.389)	0.303 (0.207, 0.380)	0.330 (0.249, 0.400)
$\boldsymbol{\gamma}$	0.432 (0.340, 0.502)	0.418 (0.325, 0.486)	0.388 (0.294, 0.460)	0.411 (0.324, 0.481)
$\boldsymbol{\delta}$	0.103 (0.063, 0.137)	0.102 (0.067, 0.133)	0.104 (0.067, 0.135)	0.103 (0.066, 0.133)
$\boldsymbol{\rho}$	1.106×10^{-3} (3.903×10^{-4}, 1.820×10^{-3})	1.199×10^{-3} (5.904×10^{-4}, 1.744×10^{-3})	1.097×10^{-3} (3.941×10^{-4}, 1.851×10^{-3})	1.160×10^{-3} (5.757×10^{-4}, 1.688×10^{-4})
$\boldsymbol{\epsilon}$	1.545×10^{-3} (1.493×10^{-4}, 3.441×10^{-3})	1.659×10^{-3} (1.465×10^{-4}, 3.732×10^{-3})	1.630×10^{-3} (1.586×10^{-4}, 3.562×10^{-3})	1.648×10^{-3} (1.324×10^{-4}, 3.587×10^{-3})
$\boldsymbol{\psi}$	0.0222 (0.0182, 0.0253)	0.0222 (0.0183, 0.0253)	0.0224 (0.0187, 0.0254)	0.0223 (0.0184, 0.0254)
**Specificity**	0.946 (0.934, 0.954)	0.957 (0.941, 0.969)	0.931 (0.909, 0.945)	0.931 (0.904, 0.949)
**Sensitivity**	0.632 (0.540, 0.699)	0.635 (0.543, 0.704)	0.644 (0.548, 0.708)	0.640 (0.549, 0.704)

medRxiv preprint doi: https://doi.org/10.1101/2023.01.06.23284260; this version posted November 28, 2023. The copyright holder for this preprint (which was not certified by peer review) is the author/funder, who has granted medRxiv a license to display the preprint in perpetuity.

It is made available under a CC-BY 4.0 International license .

Table S4. **The prior distribution specifications for the SVI_2RT model.** Parameters were given Gamma prior distributions, with hyper-parameters (a, b) listed in the table below.

**Gamma (a, b)**	β	$\tilde{\beta}$	γ	δ	ρ	ε	ψ	**Specificity**	**Sensitivity**
a	40.97	40.97	21.80	24.29	5.57	1.74	112.5	21.7	71
b	92.32	92.32	90.32	232.00	4648	1039.09	5035.15	3.83	38.3

medRxiv preprint doi: https://doi.org/10.1101/2023.01.06.23284260; this version posted November 28, 2023. The copyright holder for this preprint (which was not certified by peer review) is the author/funder, who has granted medRxiv a license to display the preprint in perpetuity.

It is made available under a CC-BY 4.0 International license .

Table S5. **Summary of the Bayesian broken stick regression results in sewersheds of Jefferson County, KY (USA).** Dispersion (σ) is the standard deviation of the error term of the linear regression.

Sewershed	Parameters	Linear regression model<br>Posterior mean (95% credible interval)
Jefferson County Aggregated	Intercept	-4.222×10^{-4} (-9.458×10^{-4}, 7.921×10^{-5})
	Alpha variant	0.815 (-0.023, 1.717)
	Delta variant	0.385 (0.318, 0.455)
	Dispersion (σ)	6.483×10^{-4} (4.543×10^{-4}, 9.490×10^{-4})
MSD1	Intercept	-7.012×10^{-4} (-1.385×10^{-3}, 1.493×10^{-5})
	Alpha variant	1.126 (0.096, 2.112)
	Delta variant	0.240 (0.181, 0.296)
	Dispersion (σ)	8.153×10^{-4} (5.739×10^{-4}, 1.186×10^{-3})
MSD2	Intercept	-2.099×10^{-4} (-8.447×10^{-4}, 4170×10^{-4})
	Alpha variant	0.881 (-0.325, 2.073)
	Delta variant	0.557 (0.482, 0.631)
	Dispersion (σ)	8.939×10^{-4} (6.330×10^{-4}, 1.300×10^{-3})
MSD3–5	Intercept	-2.963×10^{-4} (-7.426×10^{-4}, 1.508×10^{-4})
	Alpha variant	0.630 (-0.155, 1.434)
	Delta variant	0.201 (0.163, 0.240)
	Dispersion (σ)	5.635×10^{-4} (3.961×10^{-4}, 8.323×10^{-4})

medRxiv preprint doi: https://doi.org/10.1101/2023.01.06.23284260; this version posted November 28, 2023. The copyright holder for this preprint (which was not certified by peer review) is the author/funder, who has granted medRxiv a license to display the preprint in perpetuity.

It is made available under a CC-BY 4.0 International license .

Table S6. **Sensitivity analysis.** The transmission rates of the Delta variant, denoted as β*, set to 120%, 150%, 200%, 250%, and 300% of the transmission rate of the Alpha variant, denoted as β. The second column represents the corresponding increases in the basic reproduction numbers.

**Increasing amount of transmission rate of Delta variant**	$\boldsymbol{R_0}$
1.2	1.06
1.5	1.33
2.0	1.78
2.5	2.22
3.0	2.67

medRxiv preprint doi: https://doi.org/10.1101/2023.01.06.23284260; this version posted November 28, 2023. The copyright holder for this preprint (which was not certified by peer review) is the author/funder, who has granted medRxiv a license to display the preprint in perpetuity.

It is made available under a CC-BY 4.0 International license .

Table S7. **Summary of the effects of the vaccination and Delta variant in sewersheds of Jefferson County, KY (USA).** Percentage reduction due to vaccination effect or excess due to Delta variant on estimates of wastewater concentration and incidence rate. In parenthesis, we give lower and upper bounds of 95% credible interval.

		Jefferson County Aggregated	MSD1	MSD2	MSD3–5
Vaccination effect with Delta variant	Wastewater	218.9 (193.5, 242.4)	123.1 (105.0, 144.0)	202.8 (192.8, 203.4)	166.9 (146.8, 187.1)
	Incidence	156.2 (95.2, 175.7)	99.4 (94.2, 108.5)	154.5 (3.2, 154.7)	108.8 (52.8, 109.2)
Vaccination effect without Delta variant	Wastewater	44.1 (36.0, 49.9)	81.5 (77.6, 86.1)	5.7 (2.7, 12.6)	102.0 (66.5, 142.8)
	Incidence	60.3 (22.8, 62.8)	96.9 (25.2, 107.4)	36.3 (4.0, 37.7)	113.3 (14.0, 117.5)
Delta variant effect with vaccination	Wastewater	88.4 (87.7, 88.7)	82.4 (81.4, 84.0)	89.7 (88.5, 90.8)	88.3 (87.3, 89.1)
	Incidence	95.75 (95.74, 95.91)	96.78 (95.54, 96.84)	95.8 (2.7, 96.0)	97.0 (38.6, 97.1)
Delta variant effect without vaccination	Wastewater	94.5 (93.3, 95.3)	85.7 (83.7, 87.9)	95.8 (94.9, 96.7)	91.0 (90.7, 91.2)
	Incidence	97.6 (34.0, 97.7)	96.9 (8.2, 97.0)	98.0 (0.5, 98.0)	97.0 (1.1, 97.1)

medRxiv preprint doi: https://doi.org/10.1101/2023.01.06.23284260; this version posted November 28, 2023. The copyright holder for this preprint (which was not certified by peer review) is the author/funder, who has granted medRxiv a license to display the preprint in perpetuity.

It is made available under a CC-BY 4.0 International license .

Table S8. **Summary of the effects of the vaccination and Delta variant in sewersheds of Jefferson County, KY (USA).** The absolute values of difference between estimated wastewater concentrations and incidences due to vaccination effect or Delta variant. In parenthesis, we give lower and upper bounds of the 95% credible interval.

		Jefferson County Aggregated	MSD1	MSD2	MSD3–5
Vaccination effect with Delta variant	Wastewater	0.419 (0.377, 0.463)	0.266 (0.236, 0.296)	0.486 (0.456, 0.518)	0.250 (0.223, 0.277)
	Incidence	0.410 (0.035, 0.837)	0.382 (0.033, 0.797)	0.301 (0.000, 0.603)	0.413 (0.001, 0.826)
Vaccination effect without Delta variant	Wastewater	0.010 (0.008, 0.013)	0.033 (0.030, 0.035)	0.002 (0.001, 0.004)	0.019 (0.014, 0.024)
	Incidence	0.006 (0.000, 0.012)	0.011 (0.000, 0.023)	0.003 (0.000, 0.005)	0.012 (0.000, 0.025)
Delta variant effect with vaccination	Wastewater	0.182 (0.180, 0.185)	0.190 (0.183, 0.197)	0.233 (0.230, 0.237)	0.142 (0.140, 0.143)
	Incidence	0.246 (0.035, 0.456)	0.372 (0.033, 0.711)	0.187 (0.000, 0.374)	0.362 (0.001, 0.724)
Delta variant effect without vaccination	Wastewater	0.583 (0.541, 0.628)	0.440 (0.412, 0.470)	0.654 (0.625, 0.684)	0.384 (0.362, 0.406)
	Incidence	0.642 (0.001, 1.82)	0.739 (0.000, 1.478)	0.486 (0.000, 0.972)	0.767 (0.000, 1.534)

medRxiv preprint doi: https://doi.org/10.1101/2023.01.06.23284260; this version posted November 28, 2023. The copyright holder for this preprint (which was not certified by peer review) is the author/funder, who has granted medRxiv a license to display the preprint in perpetuity.

It is made available under a CC-BY 4.0 International license .

Table S9. **Vaccination effect of the incidence estimation of Jefferson County, KY (USA).** The absolute values of differences between cumulative number of the estimated incidences due to vaccination effect or Delta variant effect0. In parenthesis, we give lower and upper bounds of 95% credible interval. For comparison between sewershed zone, we estimated the incidence per 10^5 population.

		Jefferson County Aggregated	MSD1	MSD2	MSD3–5
Incidence	Vaccination	40,085 (3,507, 83,678)	38,205 (3,264, 79,673)	30,146 (1, 60,293)	40,663 (70, 81,361)
	Delta variant	24,567 (3,534, 45,601)	37,210 (3,309, 71,111)	18,693 (1, 37385)	36,210 (70, 72,351)

medRxiv preprint doi: https://doi.org/10.1101/2023.01.06.23284260; this version posted November 28, 2023. The copyright holder for this preprint (which was not certified by peer review) is the author/funder, who has granted medRxiv a license to display the preprint in perpetuity.

It is made available under a CC-BY 4.0 International license .

Table S10. **Correlation coefficients and 95% credible intervals between wastewater concentration and the estimated prevalence from the Alpha variant mutation in sewersheds of Jefferson County, KY (USA).**

	Jefferson County Aggregated	MSD1	MSD2	MSD3–5
Incidence Vaccination	0.51185 (-0.3296, 0.9427)	0.5773 (-0.2731, 0.9431)	0.9105 (0.6217, 0.9914)	0.1243 (-0.6859, 0.8364)

medRxiv preprint doi: https://doi.org/10.1101/2023.01.06.23284260; this version posted November 28, 2023. The copyright holder for this preprint (which was not certified by peer review) is the author/funder, who has granted medRxiv a license to display the preprint in perpetuity.

It is made available under a CC-BY 4.0 International license .

Table S11. **Simple linear regression model for the hospitalization rate on the observed weekly average of wastewater concentration.**

Response	Parameters	Estimate	Std.	t statistic	P-value
Hospitalization rate	Intercept	1.284×10^{-4}	2.729×10^{-5}	4.705	0.0002
	Wastewater concentration	0.1762	0.0119	14.835	0.0000

medRxiv preprint doi: https://doi.org/10.1101/2023.01.06.23284260; this version posted November 28, 2023. The copyright holder for this preprint (which was not certified by peer review) is the author/funder, who has granted medRxiv a license to display the preprint in perpetuity.

It is made available under a CC-BY 4.0 International license .

Table S12. **A simulation study summary for hierarchical regression** Each regression model was fitted using random sample data. Sample portions considered are: 100%, 83% 67%, 50%, and 33%.

Percentage	R^2	F statistics	P-value
100	0.9000	145.5	9.273×10^{-10}
83	0.8842	99.26	1.878×10^{-7}
67	0.8411	52.93	2.679×10^{-5}
50	0.7735	23.90	1.775×10^{-3}
33	0.2095	1.06	0.3614

medRxiv preprint doi: https://doi.org/10.1101/2023.01.06.23284260; this version posted November 28, 2023. The copyright holder for this preprint (which was not certified by peer review) is the author/funder, who has granted medRxiv a license to display the preprint in perpetuity.

It is made available under a CC-BY 4.0 International license .

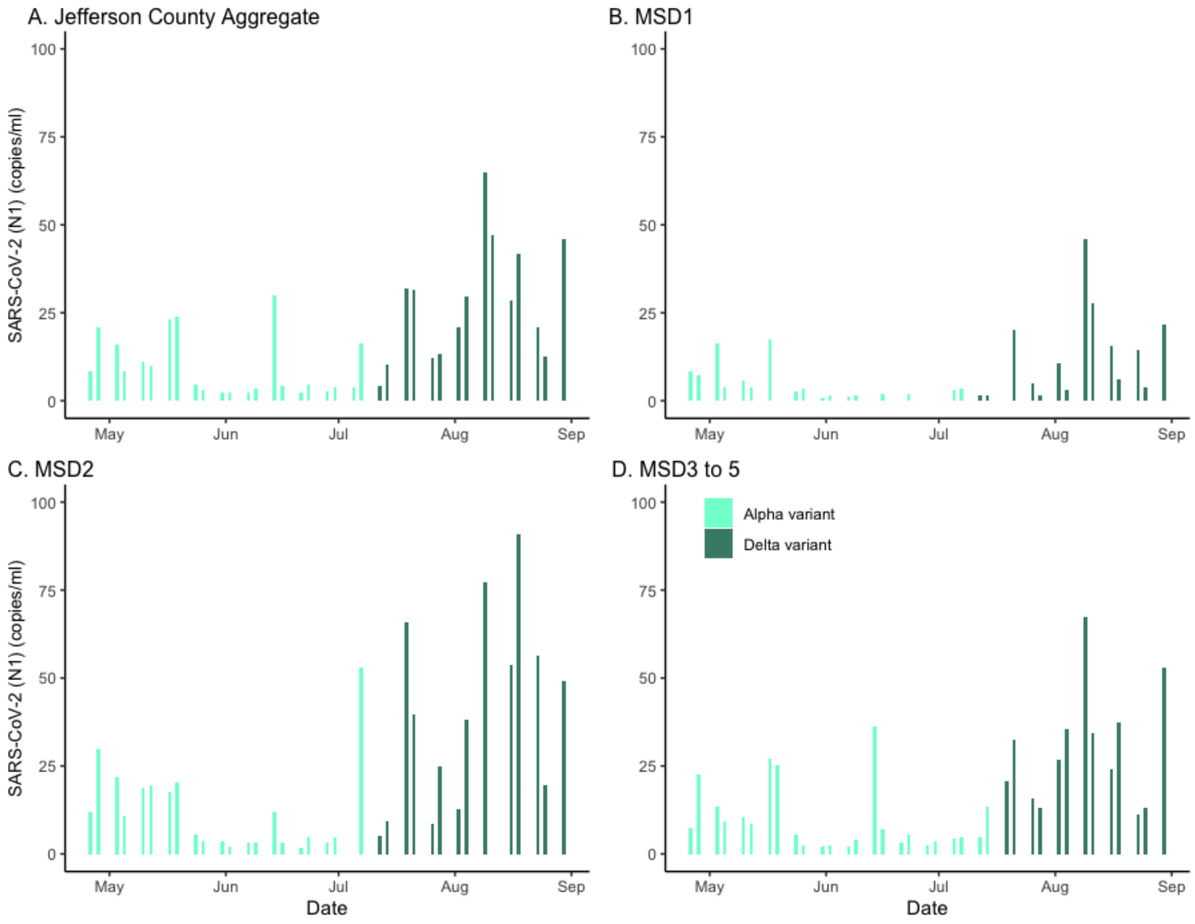

Figure S2. **SARS-CoV-2 (N1) wastewater concentration in sewersheds of Jefferson County, KY (USA).** The wastewater concentrations during Alpha and Delta variants are represented in bars (light green for Alpha variant, dark green for Delta variant). The panels compare aggregated concentration for Jefferson County (Panel A) as well as stratified by sewershed (Panels B–D).

medRxiv preprint doi: https://doi.org/10.1101/2023.01.06.23284260; this version posted November 28, 2023. The copyright holder for this preprint (which was not certified by peer review) is the author/funder, who has granted medRxiv a license to display the preprint in perpetuity.

It is made available under a CC-BY 4.0 International license .

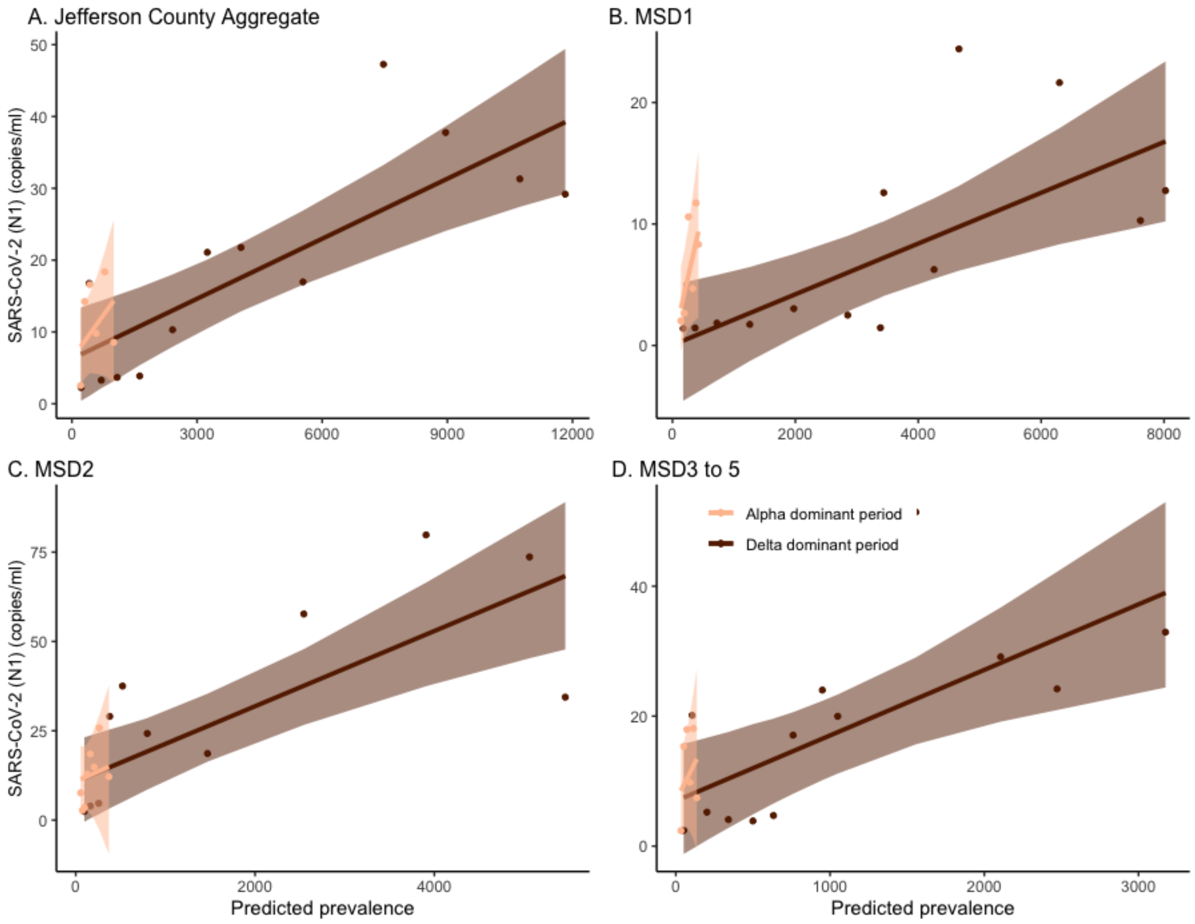

Figure S3. **Prevalence versus SARS-CoV-2 (N1) wastewater concentration in sewersheds of Jefferson County, KY (USA).** Bayesian regression between predicted weekly prevalence of SARS-CoV-2 infections from the Alpha and Delta variants and wastewater in the entire Jefferson County (Panel A) as well as stratified by sewershed (Panels B–D). The darker straight line is the fitted Bayesian regression line for the Delta variant. The darker shade marks the 95% credible interval: the lighter line and shade mark for the Alpha variant. The data points for the Alpha variant are minor (6 for Panels A, B, and D, 8 for C).

medRxiv preprint doi: https://doi.org/10.1101/2023.01.06.23284260; this version posted November 28, 2023. The copyright holder for this preprint (which was not certified by peer review) is the author/funder, who has granted medRxiv a license to display the preprint in perpetuity.

It is made available under a CC-BY 4.0 International license .

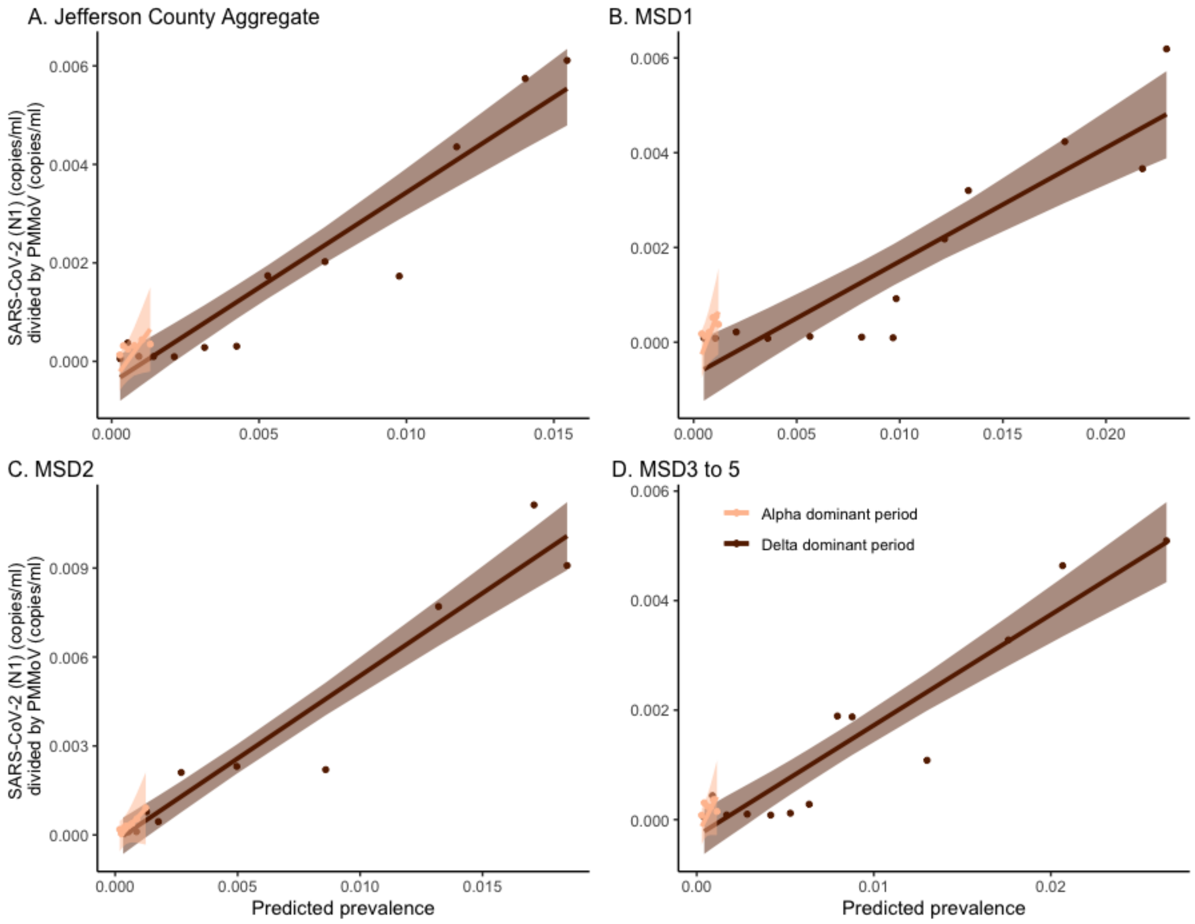

Figure S4. **Prevalence versus wastewater SARS-CoV-2 (N1) normalized by pepper mild mottle virus concentration in sewersheds of Jefferson County, KY (USA).** Bayesian regression between predicted weekly prevalence of SARS-CoV-2 infections from the Alpha and Delta variants and wastewater in the entire Jefferson County (Panel A) as well as stratified by sewershed (Panels B–D). The darker straight line is the fitted Bayesian regression line for the Delta variant. The darker shade marks the 95% credible interval: the lighter line and shade mark for the Alpha variant. The data points for the Alpha variant are very few (6 for Panels A, B, and D, 8 for C).

medRxiv preprint doi: https://doi.org/10.1101/2023.01.06.23284260; this version posted November 28, 2023. The copyright holder for this preprint (which was not certified by peer review) is the author/funder, who has granted medRxiv a license to display the preprint in perpetuity.

It is made available under a CC-BY 4.0 International license .

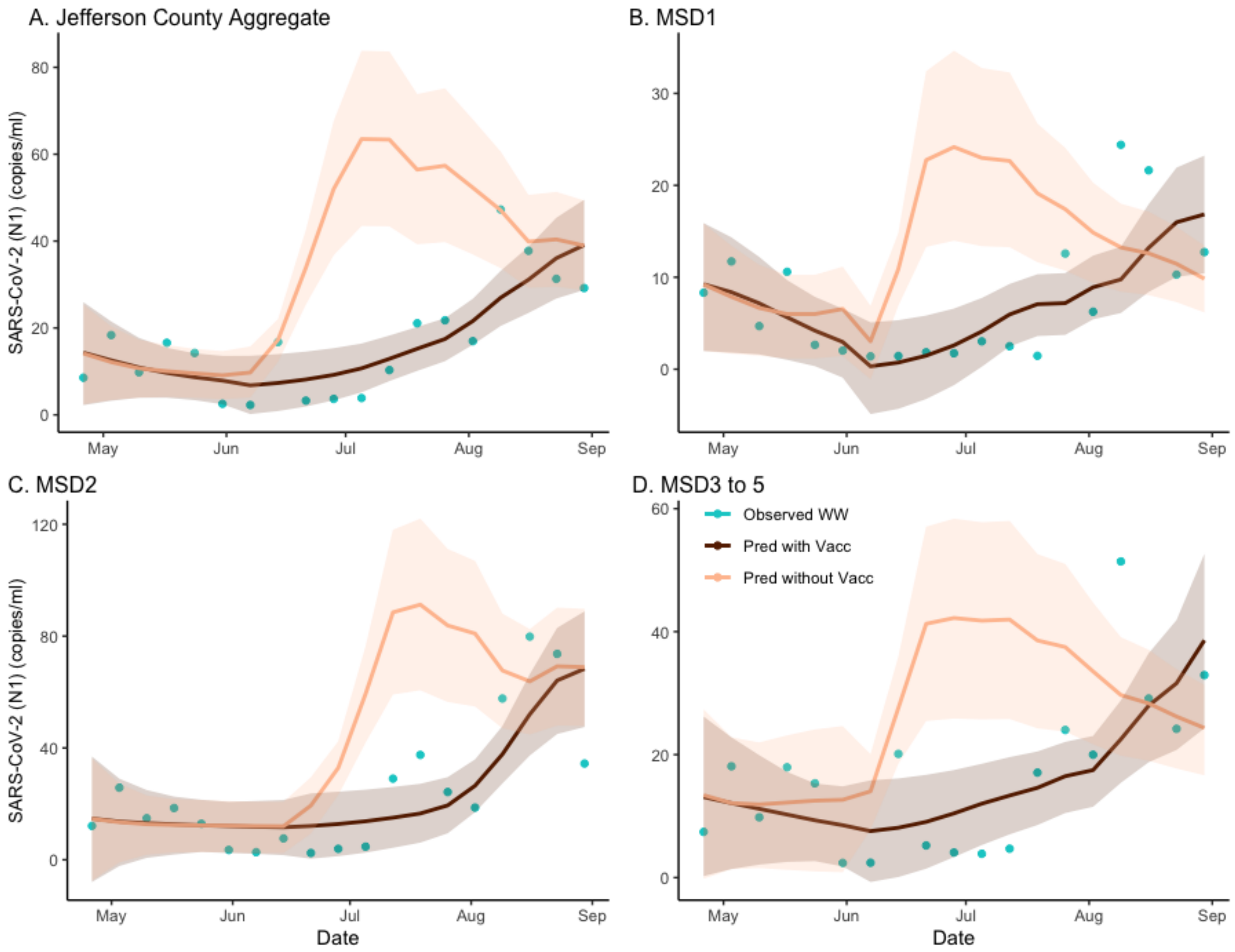

Figure S5. **The estimated effect of vaccination on SARS-CoV-2 (N1) wastewater concentration in sewersheds of Jefferson County, KY (USA).** The dark brown line is the regression-based fit to the wastewater concentration and the light brown line is the prediction of wastewater concentration using synthetic prevalence from the model with the Delta variant effect zeroed out. The shaded areas represent 95% credible intervals. The blue dots are observed weekly average wastewater concentration. The panels compare the variant effect on wastewater concentration for Jefferson County (Panel A) as well as stratified by sewershed (Panels B–D).

medRxiv preprint doi: https://doi.org/10.1101/2023.01.06.23284260; this version posted November 28, 2023. The copyright holder for this preprint (which was not certified by peer review) is the author/funder, who has granted medRxiv a license to display the preprint in perpetuity.

It is made available under a CC-BY 4.0 International license .

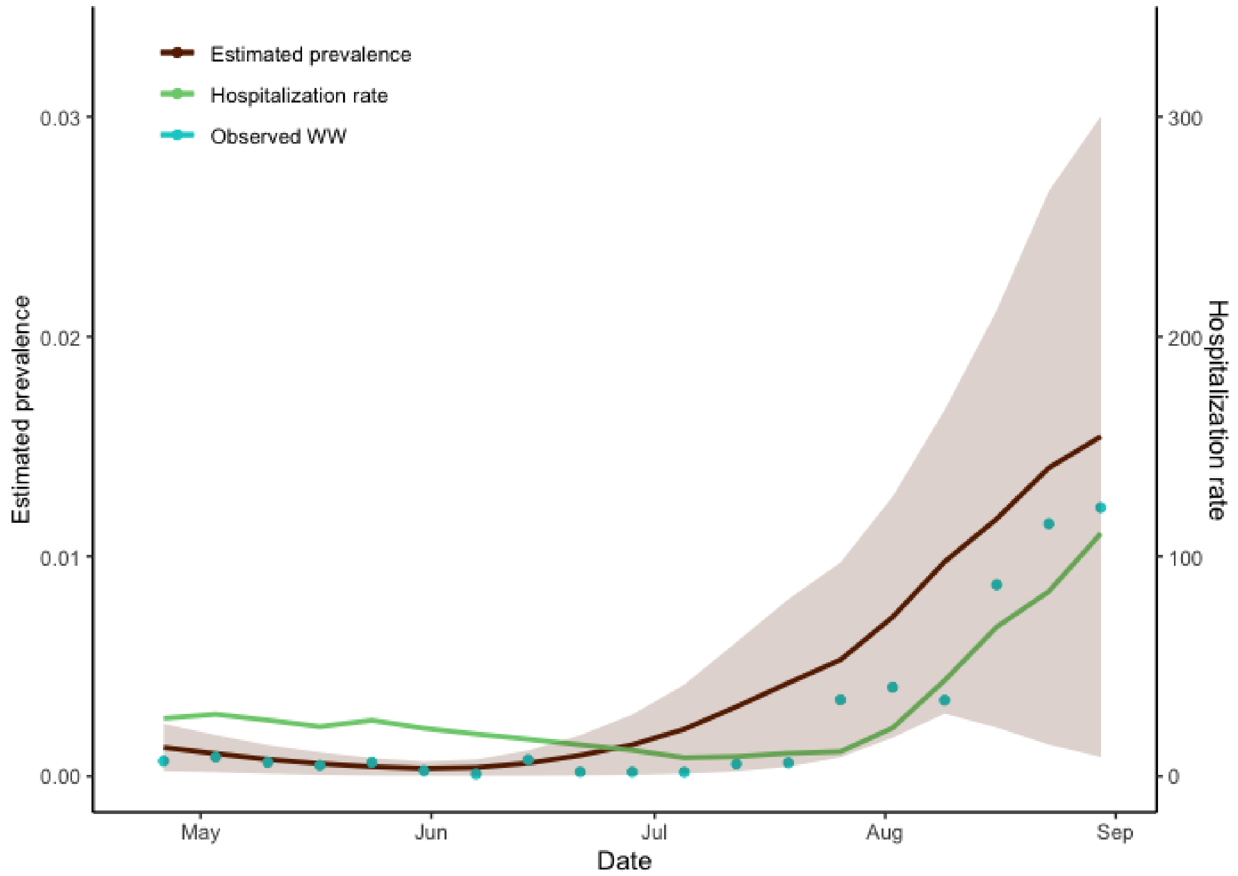

Figure S6. **SARS-CoV-2 prevalence and hospitalizations versus SARS-CoV-2 (N1) wastewater concentration normalized by pepper mild mottle virus, Jefferson County, KY (USA).** Relationship among observed wastewater concentration, the hospitalization rate, and estimated prevalence. The dark brown line represents the estimated prevalence, and the shaded area is the 95% credible interval of MCMC simulation. The green line is the weekly average of daily hospitalization rate of Jefferson County, and the blue dots represent the weekly average of wastewater concentrations. The Pearson correlation coefficient of estimated prevalence and wastewater concentration is 0.858 (95% CI = (0.502, 0.975)) and that of hospitalization rate and wastewater concentration is 0.722 (95% CI = (0.216, 0.955)).

medRxiv preprint doi: https://doi.org/10.1101/2023.01.06.23284260; this version posted November 28, 2023. The copyright holder for this preprint (which was not certified by peer review) is the author/funder, who has granted medRxiv a license to display the preprint in perpetuity.
It is made available under a CC-BY 4.0 International license .

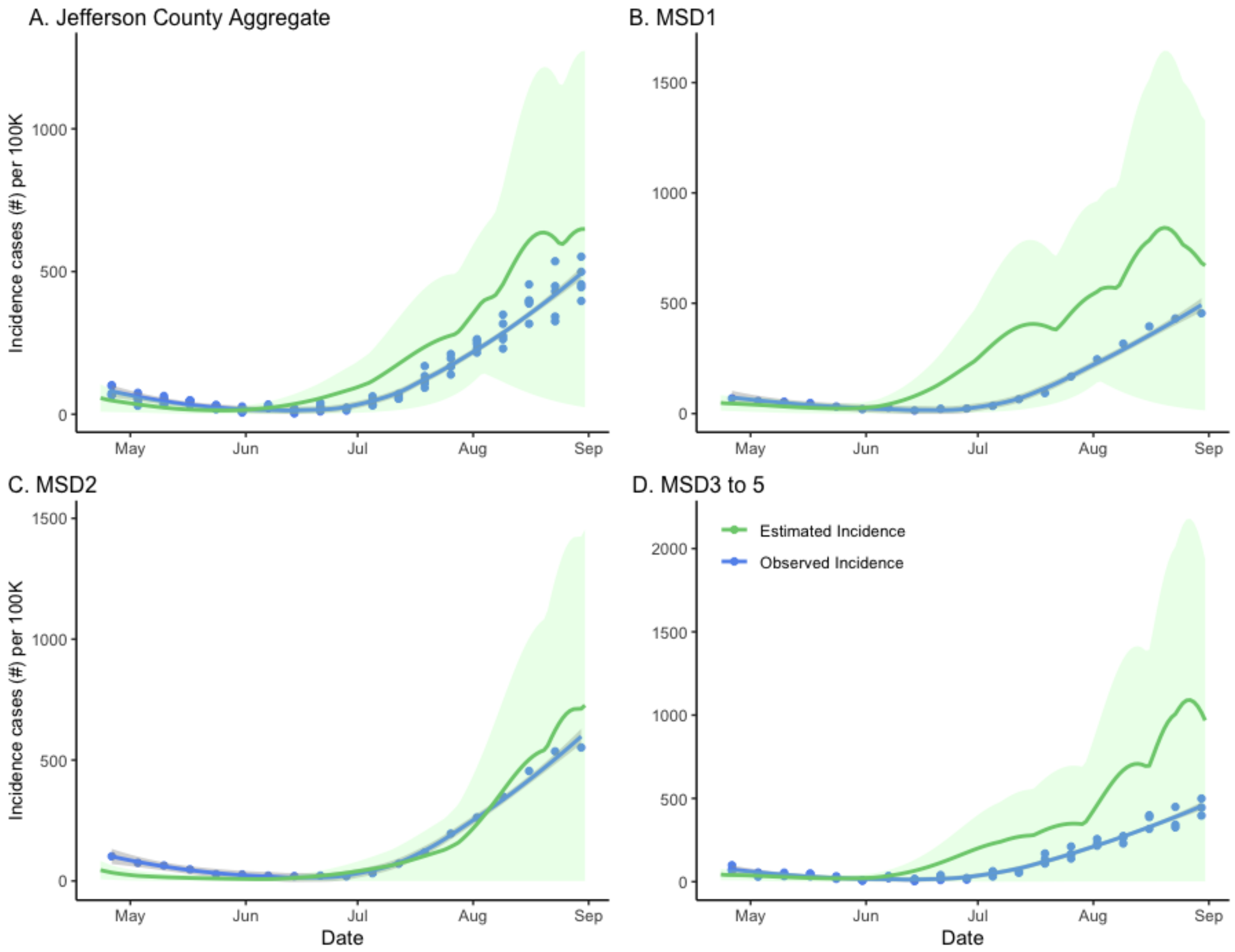

Figure S7. **Clinical versus estimated incidence in sewersheds of Jefferson County, KY (USA).** Posterior density and credibility bounds (green curve) of the weekly aggregated incidence rate as predicted by the model compared to official weekly incidence for Jefferson County (blue dots and trend line) as reported by the Jefferson County Health Department. The model plots are based on Hamiltonian MCMC samples, with 6000 steps and 2000 steps burn-in period. The panels compare aggregated incidence for Jefferson County (Panel A) as well as stratified by sewershed (Panels B–D).

medRxiv preprint doi: https://doi.org/10.1101/2023.01.06.23284260; this version posted November 28, 2023. The copyright holder for this preprint (which was not certified by peer review) is the author/funder, who has granted medRxiv a license to display the preprint in perpetuity.

It is made available under a CC-BY 4.0 International license .

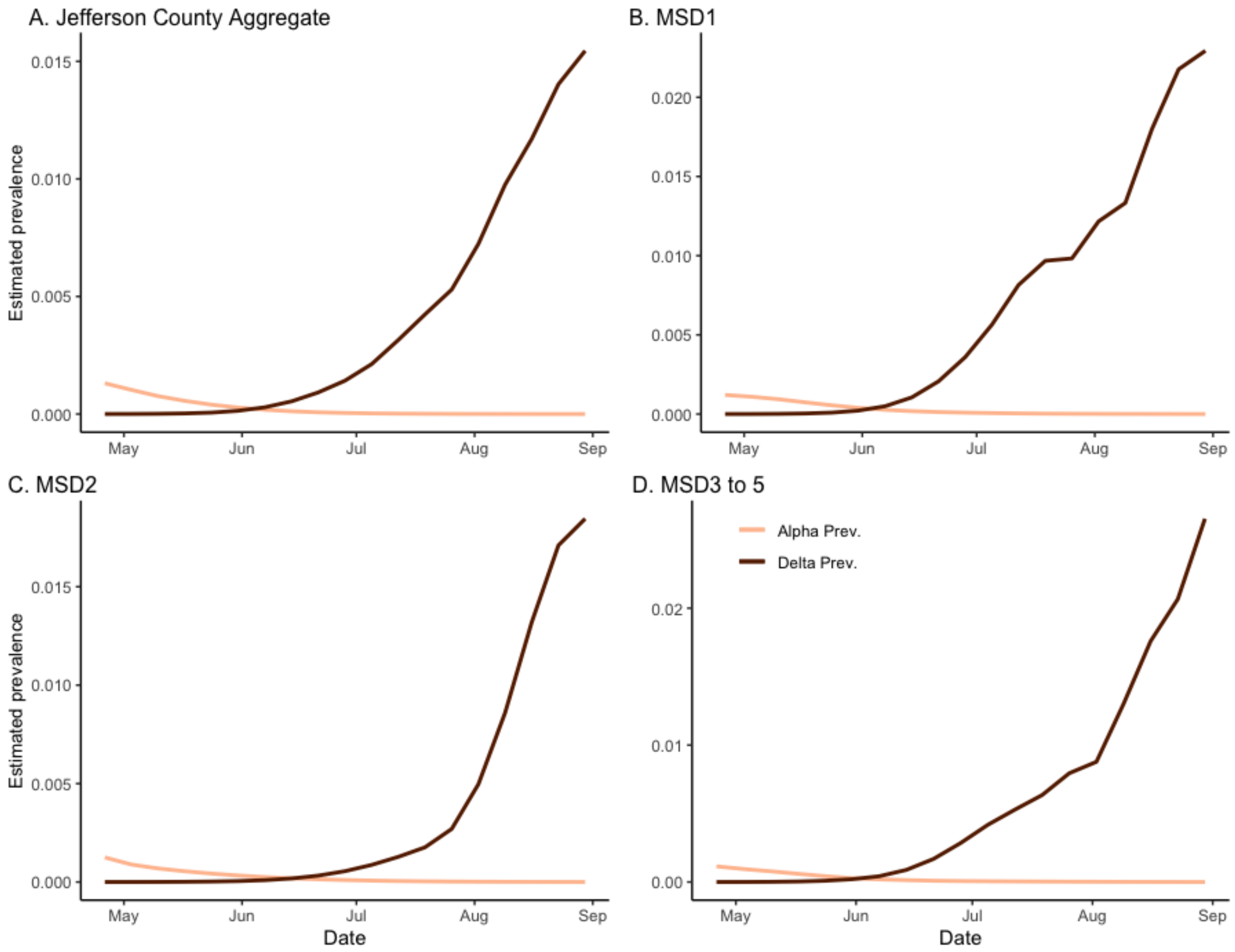

Figure S8. **Estimated prevalence for SARS-CoV-2 Alpha and Delta variants in sewersheds of Jefferson County, KY (USA).** Estimated prevalence of the Alpha and Delta variants by the model. Two estimated prevalence lines crosses at 5 June 2021 (for Panels A, B, and D) and 15 June 2021 (for Panel C), corresponding to the middle of the period of the Alpha variant being dominant. The panels compare prevalence for Jefferson County (Panel A), as well as stratified by sewershed (Panels B–D).

medRxiv preprint doi: https://doi.org/10.1101/2023.01.06.23284260; this version posted November 28, 2023. The copyright holder for this preprint (which was not certified by peer review) is the author/funder, who has granted medRxiv a license to display the preprint in perpetuity.

It is made available under a CC-BY 4.0 International license .

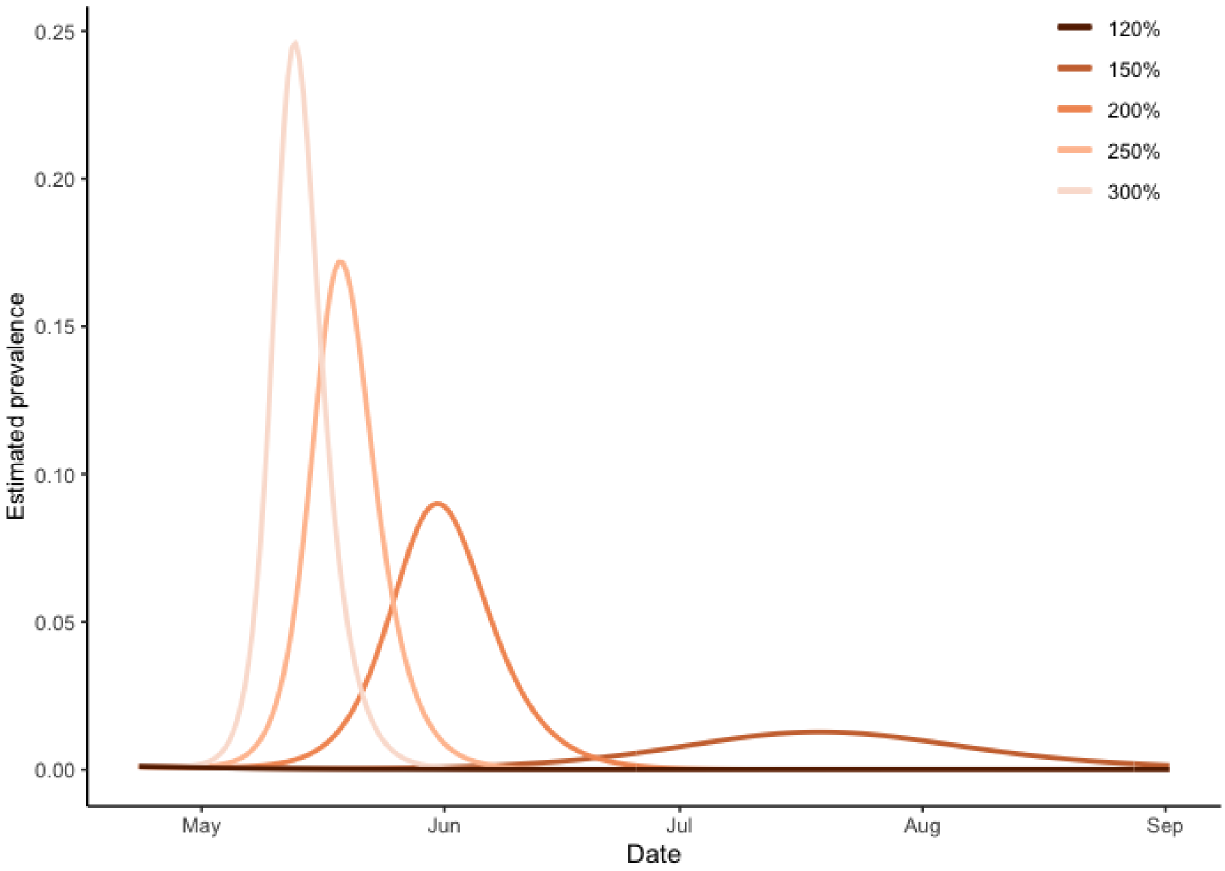

Figure S9. **Sensitivity analysis for change of prevalence according to the change of transmission rate for Delta variant.** The amount of the transmission rate for Delta variant are set from 120% to 300% which is as large as the Alpha variant transmission rate. The corresponding basic reproduction numbers are seen to change from 1.3 for 120% to 3.2 for 300%.

medRxiv preprint doi: https://doi.org/10.1101/2023.01.06.23284260; this version posted November 28, 2023. The copyright holder for this preprint (which was not certified by peer review) is the author/funder, who has granted medRxiv a license to display the preprint in perpetuity.

It is made available under a CC-BY 4.0 International license .

S3.2 Details on regression model for wastewater concentration

To relate the SVI_2RT model predictions to the serial wastewater measurements of SARS-CoV-2 (N1) concentrations and normalized SARS-CoV-2 (N1) divided by pepper mild mottle virus (PMMoV) ratio, the Bayesian linear regressions were performed based on aggregated county data and data stratified by sewershed area.

To obtain the broken stick linear regression models[5], the procedure was as follows: Let $\tilde{I}_{tj}^{(1)}$ and $\tilde{I}_{tj}^{(2)}$ be the model estimated percentage prevalence corresponding to the same week and sewershed area for the Alpha and Delta variants, respectively. We first define two basic functions $B_l(tj)$ and $B_r(tj)$:

$$B_l(tj) = \begin{cases} \tilde{I}_{ij}^{(1)} & if\ t < 06/05/2021 \\ 0 & Otherwise \end{cases},$$

and

$$B_r(tj) = \begin{cases} \tilde{I}_{ij}^{(2)} & if\ t > 06/05/2021 \\ 0 & Otherwise \end{cases}.$$

W_{tj} represents the weekly aggregated average wastewater concentration. We can now fit the model of the form:

$$W_{tj} = \beta_{0j} + \beta_{aj} B_l(tj) + \beta_{dj} B_r(tj) + e_{tj},\ e_{tj} \sim N(0, \sigma_j^2) \quad (5)$$

In the Bayesian linear regression models, non-informative priors were assigned. Specifically, the non-informative Cauchy distribution was assigned to the regression coefficients, and the non-informative gamma prior was assigned to the dispersion parameter of the error term.

S3.3 Time lag-dependency between wastewater concentration and hospitalization rate

It takes a certain period for the patient to be admitted to the hospital to receive treatment. To identify the time lag-dependency between wastewater concentration and hospitalization rate, a simple linear regression analysis was performed using a time-lagged variable as a predictor. Let W_{t-d} be the weekly aggregated average wastewater concentration at week t in the aggregated Jefferson County, and d represents a time lag. H_t represents the hospitalization rate at time t. The regression model with time lag dependent variable is given by:

$$H_t = \beta_0 + \beta_1 W_{t-d} + e_t,\ e_t \sim N(0, \sigma^2) \quad (6)$$

In this model, we changed the time lag d from 1 to 4 so that the maximum period from evidence of the community spread of COVID-19 in wastewater to reach a burden to hospitalization is about a month. Of note, hospitalizations data is available daily while wastewater is at a frequency of bi-weekly.

Additionally, we performed a simulation study using this regression model to check how much the hospitalization rate changes according to the vaccination rate. We changed the vaccination rate so that the vaccination percentage of the community was 0% and predicted the serial estimates Pred_t in Eq. (4). And then, we predicted the wastewater concentration using a linear regression model (5) and used that as the predictor in the regression model (6).

medRxiv preprint doi: https://doi.org/10.1101/2023.01.06.23284260; this version posted November 28, 2023. The copyright holder for this preprint (which was not certified by peer review) is the author/funder, who has granted medRxiv a license to display the preprint in perpetuity.

It is made available under a CC-BY 4.0 International license .

S3.4 Calculation of effects based on factual and counterfactual scenarios:

Effects of the factual and counterfactual (zero vaccinated or no Delta variant) are calculated using the area under the respective curves based on the models using factual (empirical) data and counterfactual (synthetic) data. The equation to estimate the effect is given as:

$$\left|\frac{\text{Area under counterfacutual model data}}{\text{Area under factul model data}} - 1\right|$$

medRxiv preprint doi: https://doi.org/10.1101/2023.01.06.23284260; this version posted November 28, 2023. The copyright holder for this preprint (which was not certified by peer review) is the author/funder, who has granted medRxiv a license to display the preprint in perpetuity.

It is made available under a CC-BY 4.0 International license .

S3.5 Sensitivity analysis for changing the amount of Delta variant transmission rate
In our analysis, we assumed the transmission rate of the Delta variant, denoted as β^, is 150% higher than the Alpha variant. Since this assumption is quite strong, to illustrate its effect, we conducted global sensitivity analysis under various alternative scenarios $\beta^* = \lambda\beta$,
Where β^* and β are disease transmission rates in Eq. (1). We set λ to 1.2, 1.5, 2.0, 2.5 and 3.0. Then we simulated the ODE (1) and calculate the basic reproduction number R_0.

S3.6 The derivation of the basic reproduction number (R_0)[6]
Using SVI_2RT model, let X be the vector of infected compartments, denoted by $x = \left(I^{(1)}, I^2\right)^{\mathrm{T}}$. The system has a disease-free state $x_0 = (\mathrm{S}_0, V_0, I_0^{(1)}, I_0^{(2)}, R_0, T_0)$. We define the matrix of new infection $\mathcal{F}(x)$ and the matrix of all transitions except for the new infection V. The net transition rates are represented by $V(x)$.

$$\mathcal{F}(x) = \begin{pmatrix} \beta S_t I_t^{(1)} + \tilde{\beta} V_t I_t^{(1)} \\ \beta^* S_t I_t^{(2)} + \tilde{\beta}^* V_t I_t^{(2)} \end{pmatrix}, V(x) = \begin{pmatrix} \gamma I^{(1)} \\ \gamma I^{(2)} \end{pmatrix}$$

The next generation matrix is defined as FV^{-1} where F and V represent 2×2 matrices at x_0 as follows:

$$\mathrm{F} = \begin{bmatrix} \beta S_0 + \tilde{\beta} V_0 & 0 \\ 0 & \beta^* S_0 + \tilde{\beta}^* V_0 \end{bmatrix}, \text{ and } \mathrm{V} = \begin{bmatrix} \gamma & 0 \\ 0 & \gamma \end{bmatrix}$$

The next generation matrix K is calculated as

$$K = \mathrm{FV}^{-1} = \begin{bmatrix} \frac{\beta S_0 + \tilde{\beta} V_0}{\gamma} & 0 \\ 0 & \frac{\beta^* S_0 + \tilde{\beta}^* V_0}{\gamma} \end{bmatrix}$$

Finally, the basic reproduction number R_0 is the maximum eigenvalue of the spectral decomposition of the next generation matrix K:

$$R_0 = \frac{\beta^* S_0 + \tilde{\beta}^* V_0}{\gamma}.$$

If we set $S_0 = 1$ and $V_0 = 0$, then $R_0 = \frac{\beta^*}{\gamma}$.

medRxiv preprint doi: https://doi.org/10.1101/2023.01.06.23284260; this version posted November 28, 2023. The copyright holder for this preprint (which was not certified by peer review) is the author/funder, who has granted medRxiv a license to display the preprint in perpetuity.

It is made available under a CC-BY 4.0 International license .

References

1 Boyle, L. et al. Selective sweeps in SARS-CoV-2 variant competition. *Proc. Natl. Acad. Sci. USA* **119**(47), e2213879119 (2022). https://doi.org/10.1073/pnas.2213879119

2 Kläser, K. et al. COVID-19 due to the B.1.617.2 (Delta) variant compared to B.1.1.7 (Alpha) variant of SARS-CoV-2: a prospective observational cohort study. Sci. Rep.-UK ***12***(1), 10904 (2022). https://doi.org/10.1038/s41598-022-14016-0

3 Smith, T. et al. Quantifying the relationship between sub-population wastewater samples and community-wide SARS-CoV-2 seroprevalence. *Sci. Total Environ.* **853**, 158567 (2022). https://doi.org/10.1016/j.scitotenv.2022.158567

4 KhudaBukhsh, W. R., Choi, B., Kenah, E. & Rempala, G. A. Survival dynamical systems: individual-level survival analysis from population-level epidemic models. *Interface Focus* **10**(1), 20190048 (2020). https://doi.org/10.1098/rsfs.2019.0048

5 Faraway, J. J. *Linear models with R*. 2nd ed., CRC press (2016).

6 Van den Driessche, P. Reproduction numbers of infectious disease models. *Infectious Disease Modelling* ***2***(3), 288–303 (2017). https://doi.org/10.1016/j.idm.2017.06.002

medRxiv preprint doi: https://doi.org/10.1101/2023.01.06.23284260; this version posted November 28, 2023. The copyright holder for this preprint (which was not certified by peer review) is the author/funder, who has granted medRxiv a license to display the preprint in perpetuity.

It is made available under a CC-BY 4.0 International license .

Appendix D. Wastewater variant detection

Table S13. **Periods of Alpha and Delta variant wastewater dominance in sewersheds of Jefferson County, KY (USA).** Dates determined by shift in major variant based on sampling schedule of wastewater collection.

Sewershed	Alpha dominant in wastewater		Delta dominant in wastewater	
	Start date	End date	Start date	End date
MSD1	3/30/21	5/17/21	7/12/21	8/30/21
MSD2	3/30/21	5/24/21	7/12/21	8/30/21
MSD3	3/30/21	6/21/21	7/19/21	8/30/21
MSD4	3/30/21	7/5/21	7/19/21	8/30/21
MSD5	3/30/21	6/28/21	7/26/21	8/30/21
