# Supplementary material for: SARS-CoV-2 wastewater concentration and linked longitudinal seroprevalence: a spatial analysis of strain mutation, post-COVID-19 vaccination effect, and hospitalization burden forecasting
Source: medRxiv. 2023 Jan 7:2023.01.06.23284260. Preprint. [Version 1] doi: 10.1101/2023.01.06.23284260 (PMC9844017; doi:10.1101/2023.01.06.23284260)
Supplement: Supplementary file 1 [file NIHPP2023.01.06.23284260V1-supplement-1.pdf]

medRxiv preprint doi: https://doi.org/10.1101/2023.01.06.23284260; this version posted January 7, 2023. The copyright holder for this preprint (which was not certified by peer review) is the author/funder, who has granted medRxiv a license to display the preprint in perpetuity.
It is made available under a CC-BY 4.0 International license .

Supplementary Material

medRxiv preprint doi: https://doi.org/10.1101/2023.01.06.23284260; this version posted January 7, 2023. The copyright holder for this preprint (which was not certified by peer review) is the author/funder, who has granted medRxiv a license to display the preprint in perpetuity.
It is made available under a CC-BY 4.0 International license .

Table of Contents

S1. SARS-CoV-2 seroprevalence by wave and sewershed, Jefferson County, KY (USA).......... 23

S2. Studied wastewater treatment plant zones (sewersheds), Jefferson County, KY (USA). 24

S3. Population vaccination model (SVI$_2$RT)... 26

S4. Variant detection.. 40

medRxiv preprint doi: https://doi.org/10.1101/2023.01.06.23284260; this version posted January 7, 2023. The copyright holder for this preprint (which was not certified by peer review) is the author/funder, who has granted medRxiv a license to display the preprint in perpetuity.
It is made available under a CC-BY 4.0 International license .

S1. SARS-CoV-2 seroprevalence by wave and sewershed, Jefferson County, KY (USA).

Table S1. **SARS-CoV-2 seroprevalence by wave and sewershed, Jefferson County, KY (USA).**

	Number of unvaccinated participants	Number of vaccinated participants	Number of participants positive for SARS-CoV-2 nucleocapsid (N) specific IgG antibodies	Estimated posterior average seroprevalence per 10^5 people (95% credible interval	Estimated posterior average prevalence per 10^5people (95% credible interval)
Overall					
MSD1	91	1450	132	9153 (3772, 14533)	533 (38, 1028)
MSD2	132	794	81	5427 (1848, 9006)	336 (0, 672)
MSD3-5	23	228	17	8410 (2016, 14804)	588 (0, 1177)
Total	315	2957	296	22989 (13898, 32081)	1457 (618, 2296)
Wave A					
MSD1	26	363	24	2249 (1686, 2811)	62 (1, 122)
MSD2	29	206	25	2277 (1751, 2803)	49 (0, 97)
MSD3-5	4	39	5	2284 (1682, 2887)	53 (1, 106)
Total	68	697	68	6810 (5832, 7788)	163 (70, 257)
Wave B					
MSD1	20	369	17	2927 (2042, 3813)	197 (7, 386)
MSD2	40	192	17	2611 (1832, 3391)	74 (0, 149)
MSD3-5	7	53	2	2952 (1816, 4089)	271 (0, 542)
Total	89	730	42	8491 (6853, 10129)	542(203, 881)
Wave C					
MSD1	16	308	22	6261 (2537, 9984)	618 (43, 1193)
MSD2	29	179	11	3696 (1860, 5531)	197 (0, 394)
MSD3-5	3	60	2	7565 (2048, 13083)	456 (0, 911)
Total	60	657	46	17521 (10617, 24426)	1270 (511, 2030)
Wave D					
MSD1	29	410	69	19572 (8999, 30145)	810 (4, 1617)
MSD2	34	217	28	12408 (1872, 22943)	834(0, 1668)
MSD3-5	9	76	8	14746 (2188, 27304)	1440 (0, 2880)
Total	98	873	140	46726 (27220, 66232)	3084 (1235, 4934)

medRxiv preprint doi: https://doi.org/10.1101/2023.01.06.23284260; this version posted January 7, 2023. The copyright holder for this preprint (which was not certified by peer review) is the author/funder, who has granted medRxiv a license to display the preprint in perpetuity.
It is made available under a CC-BY 4.0 International license .

S2. Studied wastewater treatment plant zones (sewersheds), Jefferson County, KY (USA).

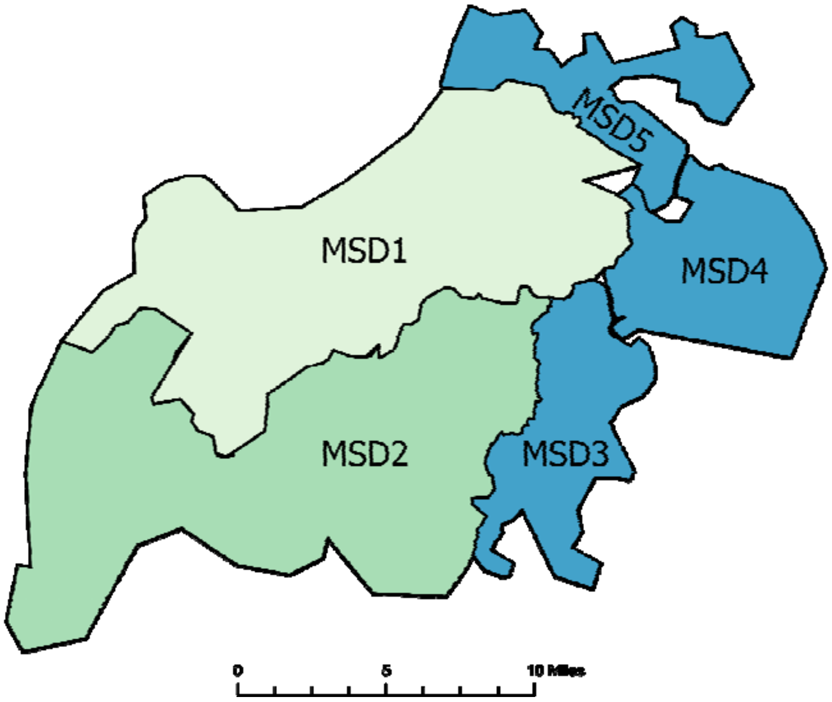

Figure S1. **Studied wastewater treatment plant sewersheds, Jefferson County, Kentucky (USA).**

medRxiv preprint doi: https://doi.org/10.1101/2023.01.06.23284260; this version posted January 7, 2023. The copyright holder for this preprint (which was not certified by peer review) is the author/funder, who has granted medRxiv a license to display the preprint in perpetuity.
It is made available under a CC-BY 4.0 International license .

Table S2. **Characteristics of studied wastewater treatment plant sewersheds of Jefferson County, KY (USA).**

Site name	Income (USD$)[a]	Population[a]	Area (km^2)	Combined sewer
MSD01<br>Morris Forman Water Quality Treatment Center (MFWQTC)	54,138	349,850	280	Yes
MSD02<br>Derek R. Guthrie Water Quality Treatment Center (DRGWQTC)	53,577	295,910	332	No
MSD03<br>Cedar Creek Water Quality Treatment Center (CCWQTC)	76,606	55,928	80	No
MSD04<br>Floyds Fork Water Quality Treatment Center (FFWQTC)	113,699	32,460	88	No
MSD05<br>Hite Creek Water Quality Treatment Center (HCWQTC)	106,769	31,269	67	No

[a] Based on 2018 U.S Census Bureau American Community Survey (ACS). Income is mean median household.

medRxiv preprint doi: https://doi.org/10.1101/2023.01.06.23284260; this version posted January 7, 2023. The copyright holder for this preprint (which was not certified by peer review) is the author/funder, who has granted medRxiv a license to display the preprint in perpetuity.
It is made available under a CC-BY 4.0 International license .

S3. Population vaccination model (SVI_2RT)

The equation shown in (1) describes the time-evolution of the proportions of individuals who are susceptible (S), vaccinated (V), infected with Alpha variant(I_1), infected with Delta variant (I_2) removed (R), and seropositive (T). We assume the total initial population of susceptibles is large with a small initial fraction of infected. The model equations are

$$\begin{aligned} \dot{S}_t &= -\beta S_t I_t^{(1)} - \beta^* S_t I_t^{(2)} - \alpha_t S_t, \\ \dot{V}_t &= \alpha_t S_t - \tilde{\beta} I_t^{(1)} V_t - \tilde{\beta}^* I_t^{(2)} V_t \\ \dot{I}_t^{(1)} &= \beta S_t I_t^{(1)} + \tilde{\beta} I_t^{(1)} V_t - \gamma I_t^{(1)}, \\ \dot{I}_t^{(2)} &= \beta^* S_t I_t^{(2)} + \tilde{\beta}^* I_t^{(2)} V_t - \gamma I_t^{(2)} \\ \dot{R}_t &= \gamma I_t - \delta R_t, \\ \dot{T}_t &= \delta R_t, \end{aligned} \quad (1)$$

with the initial condition $S_0 = 1 - \rho - \epsilon - \psi > 0, V_0 = 0$, $I_0^{(1)} = \rho > 0$, $I_0^{(2)} = \rho/100$, $R_0 = \epsilon > 0$, and $T_0 = \psi > 0$.

Here, β and $\tilde{\beta}$ are the rates of infection of respectively, unvaccinated and vaccinated, and β^* and $\tilde{\beta}^*$ are the rates of infection according to Delta variant. As our compartment model has two infection compartments, it is called the variant competition model.[1] The observed data in this analysis do not have any information about infection from the Delta variant, and an increase in the number of parameters makes model estimation difficult and may lead to identifiability problems. So, we set β^* and $\tilde{\beta}^*$ at the values 20% higher than β and $\tilde{\beta}$.[2] The function of α_t represents a changing rate of vaccination over time. However, the vaccination process can not follow a stochastic process but may be changed according to a policy or vaccine supply, so set the vaccination rate $\alpha(t)$ to match the empirical percentage of the vaccinated population in Jefferson County at the end of August 2021. Additionally, γ is the rate of recovery, and δ is the rate at which antibodies build to a detectable level after recovery. The SVI_2RT model parameters to be estimated are given by the vector $\theta = (\beta, \tilde{\beta}, \gamma, \delta, \rho, \epsilon, \psi)$.

To obtain the serial estimates of incidence and prevalence from the observed seropositivity levels in four waves of testing, we adapt the idea of an ODE-based survival model proposed recently.[3, 4] According to that model, the scaled quantities $S_t, V_t, I_t^{(1)}, I_t^{(2)}, R_t, T_t$ may be considered as respective probabilities of a randomly selected individual in a large population, being either susceptible, vaccinated, infected with different virus variant, recovered, or seroprevalent at time t. Consequently, we consider the results $Z(t)$ of all individual antibody-based tests conducted at times t as independent Bernoulli variables:
$Z(t) \sim \text{Ber}(T_t^*)$,
where $T_t^* = sensT_t + (1 - spe)(1 - T_t)$ is the specificity adjusted probability of a positive test. For our analysis, both $sens$ and spe are additional parameters to be estimated. We assigned the informative priors to $sens$ and spe from available clinical data.

Assuming at time t, n_t individuals are tested with k_t having positive results, the corresponding log-likelihood function is

medRxiv preprint doi: https://doi.org/10.1101/2023.01.06.23284260; this version posted January 7, 2023. The copyright holder for this preprint (which was not certified by peer review) is the author/funder, who has granted medRxiv a license to display the preprint in perpetuity.
It is made available under a CC-BY 4.0 International license .

$\ell_t(\Theta) \propto k_t \log(T_t) + (n_t - k_t)\log(1 - T_t)$, (2)

where $\Theta = (\beta, \tilde{\beta}, \gamma, \delta, \rho, \epsilon, \psi, spe, sens)$ is the vector of parameters to be identified. Given the testing data at $m \geq 1$ time points $t_1, \ldots, t_m$, we then aim to find parameter values θ that maximizes the posterior log-likelihood function

$\tilde{\ell}(\Theta) \propto \sum_{i=1}^{m} \ell_{t_i}(\Theta) + \log p(\Theta)$, (3)

where $p(\Theta)$ is the prior distribution on Θ to be determined from our previous work.[3] Hence, we seek the values of Θ that maximize our posterior log-likelihood function (3). Note the entire system (1) must be solved for each parameter combination.

S3.1 Incidence, prevalence, and seroprevalence estimation

Posterior serial estimates of the relative rates of incidence, prevalence, and seropositivity were obtained from the SVI_2RT model as the time-dependent vector

$\mathrm{Pred}_t = (-\dot{S}_t, V_t, I_t^{(1)}, I_t^{(2)}, T_t)$. (4)

Here $(S_t, V_t, I_t^{(1)}, I_t^{(2)}, T_t)$ is the family of trajectories of (1) evaluated at the posterior distribution of the vector Θ. In practice, the distribution of Pred_t is approximated by taking a random sample of size m from the converged MCMC sampler. In our case $m = 2000$. To obtain daily incidence rates (Inc_d) we have used the approximation $\dot{S}_t \approx S_{t+1} - S_t$ and consequently took $\mathrm{Inc}_d = S_d - S_{d+1}$ where d corresponds to a specific day of interest. The estimated prediction counts were obtained by multiplying the rates in Pred_t by the appropriate population numbers.

medRxiv preprint doi: https://doi.org/10.1101/2023.01.06.23284260; this version posted January 7, 2023. The copyright holder for this preprint (which was not certified by peer review) is the author/funder, who has granted medRxiv a license to display the preprint in perpetuity.
It is made available under a CC-BY 4.0 International license .

Table S3.1. **Posterior mean estimates of the SVI_2RT model parameters in sewersheds of Jefferson County, KY (USA).** The area-specific Hamiltonian Markov chain Monte Carlo (MCMC) posterior estimates are based on seropositivity data aggregated across Jefferson County and stratified by sewersheds. The corresponding 95% credible bounds are provided in parenthesis. The results are based on MCMC implemented via *Rstan* library, with a 6000- and 2000-step burn-in.

	**Jefferson County Aggregated**	**MSD1**	**MSD2**	**MSD3–5**
β	0·494 (0·389, 0·572)	0·493 (0·391, 0·575)	0·419 (0·309, 0·503)	0·459 (0·347, 0·542)
α	0·009 (64%)	0·010 (67%)	0·007 (55%)	0·013 (76%)
$\tilde{\beta}$	0·420 (0·321, 0·502)	0·423 (0·3254, 0·500)	0·412 (0·384, 0·587)	0·445 (0·335, 0·532)
γ	0·463 (0·376, 0·532)	0·453 (0·371, 0·520)	0·494 (0·384, 0·587)	0·461 (0·371, 0·533)
δ	0·104 (0·067, 0·136)	0·104 (0·066, 0·136)	0·105 (0·069, 0·138)	0·104 (0·066, 0·135)
ρ	3·750 x 10^{-4} (6·385 x 10^{-5}, 7·426 x 10^{-4})	4·781 x 10^{-4} (9·191 x 10^{-5}, 9·221 x 10^{-4})	4·382 x 10^{-4} (6·034 x 10^{-5}, 9·113 x 10^{-3})	4·494 x 10^{-4} (7·366 x 10^{-5}, 9·099 x 10^{-4})
ϵ	1·506 x 10^{-3} (1·396 x 10^{-4}, 3·471 x 10^{-3})	1·638 x 10^{-3} (1·170 x 10^{-4}, 3·618 x 10^{-3})	1·635 x 10^{-3} (1·802 x 10^{-4}, 3·739 x 10^{-3})	1·649 x 10^{-3} (1·526 x 10^{-4}, 3·619 x 10^{-3})
ψ	0·0223 (0·0182, 0·0253)	0·0222 (0·0183, 0·0253)	0·0223 (0·0184, 0·0254)	0·0223 (0·0184, 0·0254)
**Specificity**	0·946 (0·934, 0·954)	0·958 (0·941, 0·969)	0·926 (0·905, 0·941)	0·929 (0·898, 0·948)
**Sensitivity**	0·637 (0·539, 0·705)	0·638 (0·540, 0·703)	0·648 (0·556, 0·714)	0·643 (0·551, 0·711)

medRxiv preprint doi: https://doi.org/10.1101/2023.01.06.23284260; this version posted January 7, 2023. The copyright holder for this preprint (which was not certified by peer review) is the author/funder, who has granted medRxiv a license to display the preprint in perpetuity.
It is made available under a CC-BY 4.0 International license .

Table S3.2. **The prior distribution specifications for the SIRT model.** Parameters were given Gamma prior distributions, with hyper-parameters (*a*, *b*) listed in the table below.

**Gamma (a, b)**	β	$\tilde{\beta}$	γ	δ	ρ	ε	ψ	**Specificity**	**Sensitivity**
a	40·97	40·97	41·80	24·29	2·18	1·74	112·5	21·7	71
b	92·32	92·32	90·32	232·00	4648	1039·09	5035·15	3·83	38·3

Table S3.3. **Summary of the effects of the vaccination and Delta variant mutation in sewersheds of Jefferson County, KY (USA).** Percentage reduction due to vaccination effect or excess due to Delta variant on estimates of wastewater concentration (WW) and incidence rate (Incidence). In parenthesis, we give lower and upper bounds of 95% credible (incidence) or confidence interval (wastewater). Some values were rounded down to 0 for simplicity.

		Jefferson County Aggregated	MSD1	MSD2	MSD3–5
Vaccination effect with Delta variant	WW	179 (162, 200)	119 (86, 157)	262 (238, 284)	135 (124, 146)
	Incidence	133 (0, 147)	88 (0, 94)	173 (27, 173)	80 (0, 80)
Vaccination effect without Delta variant	WW	1370 (1272, 1493)	603 (479, 818)	823 (801, 849)	928 (848, 1027)
	Incidence	564 (0, 573)	323 (0, 330)	363 (0, 364)	491 (13, 491)
Delta variant effect without vaccination	WW	1157 (1049, 1291)	524 (429, 689)	1393 (1235, 1590)	741 (665, 835)
	Incidence	632 (1913, 605)	340 (664, 333)	758 (0, 760)	516 (18, 516)
Delta variant effect without vaccination	WW	159 (134, 185)	98 (73, 126)	478 (389, 581)	101 (89, 113)
	Incidence	143 (51, 144)	99 (25, 99)	308 (8, 308)	101 (0, 101)

medRxiv preprint doi: https://doi.org/10.1101/2023.01.06.23284260; this version posted January 7, 2023. The copyright holder for this preprint (which was not certified by peer review) is the author/funder, who has granted medRxiv a license to display the preprint in perpetuity.
It is made available under a CC-BY 4.0 International license .

Table S3.4. **Summary of the Bayesian regression results in sewersheds of Jefferson County, KY (USA).** Dispersion (σ) is the standard deviation of the error term of the linear regression.

Sewershed	Parameters	Linear regression model<br>Posterior mean (95% credible interval)
Jefferson County Aggregated	Intercept	-5·563 x 10^{-4} (-9·903 x 10^{-4} , -1·250 x 10^{-4})
	Slope	0·453 (0·374, 0·529)
	Dispersion (σ)	6·434 x 10^{-4} (4·589 x 10^{-4} , 9·191 x 10^{-4})
	Correlation	0·916 (0·764, 0·976)
MSD1	Intercept	-8·684 x 10^{-4} (-1·757 x 10^{-4} , 4·119 x 10^{-5})
	Slope	0·357 (0·230, 0·481)
	Dispersion (σ)	1·143 x 10^{-4} (8·237 x 10^{-4} , 1·642 x 10^{-3})
	Correlation	0·754 (0·463, 0·913)
MSD2	Intercept	-2·328 x 10^{-4} (-1·001 x 10^{-3}, 5·280 x 10^{-4})
	Slope	0·932 (0·741, 1·114)
	Dispersion (σ)	1·339 x 10^{-3} (9·603 x 10^{-4} , 1·948 x 10^{-3})
	Correlation	0·770 (0·376, 0·944)
MSD3–5	Intercept	-3·978 x 10^{-4} (-7·405 x 10^{-4} , -3·951 x 10^{-5})
	Slope	0·284 (0·236, 0·331)
	Dispersion (σ)	5·407 x 10^{-4} (3·843 x 10^{-4} , 7·697 x 10^{-4})
	Correlation	0·896 (0·542, 0·976)

medRxiv preprint doi: https://doi.org/10.1101/2023.01.06.23284260; this version posted January 7, 2023. The copyright holder for this preprint (which was not certified by peer review) is the author/funder, who has granted medRxiv a license to display the preprint in perpetuity.
It is made available under a CC-BY 4.0 International license .

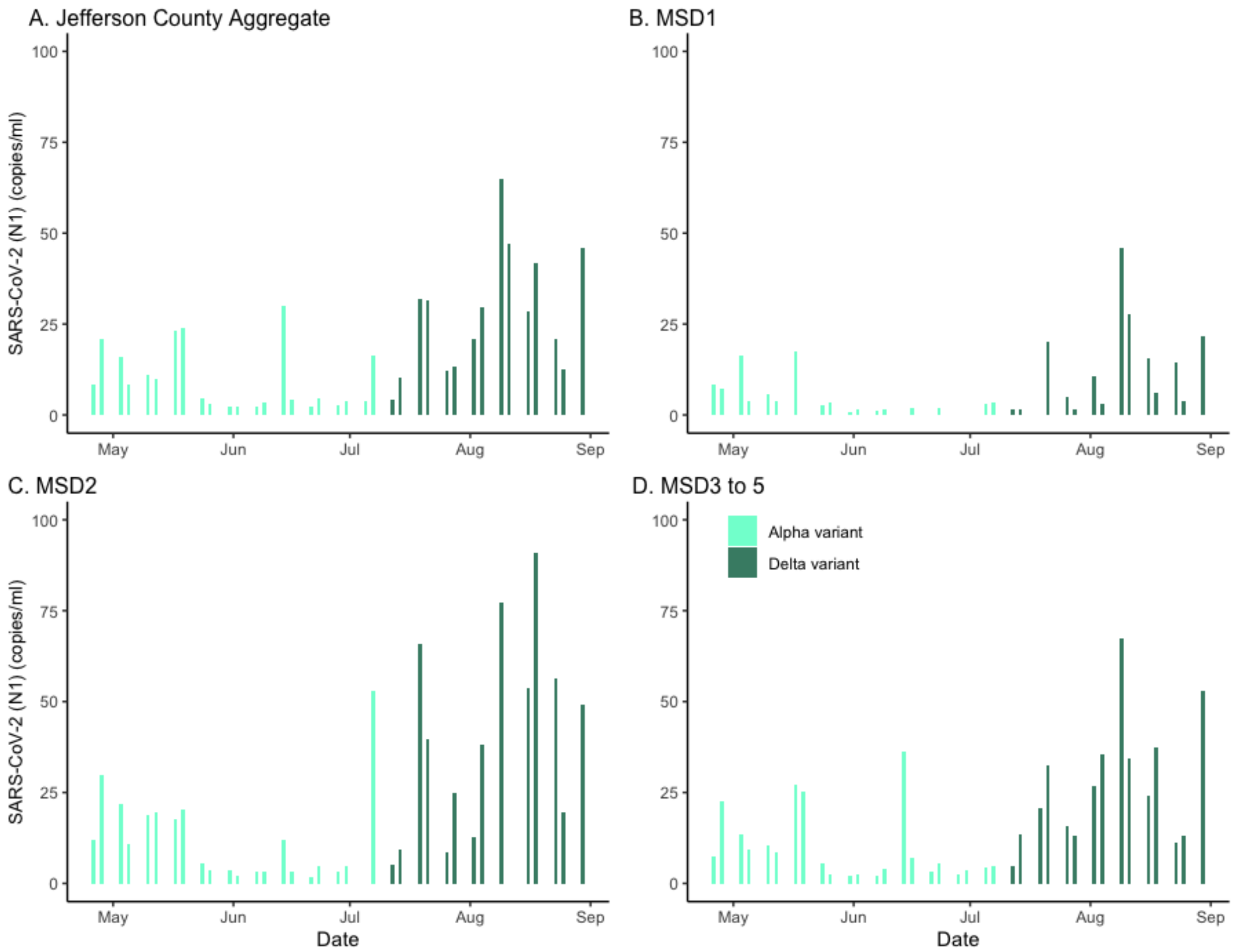

Figure S3.1. **SARS-CoV-2 (N1) wastewater concentration in sewersheds of Jefferson County, KY (USA).** The wastewater concentrations during Alpha and Delta variants are represented in bars (light green for Alpha, dark green for Delta). The panels compare aggregated concentration for Jefferson County (Panel A) as well as stratified by sewershed (Panels B–D).

medRxiv preprint doi: https://doi.org/10.1101/2023.01.06.23284260; this version posted January 7, 2023. The copyright holder for this preprint (which was not certified by peer review) is the author/funder, who has granted medRxiv a license to display the preprint in perpetuity.
It is made available under a CC-BY 4.0 International license .

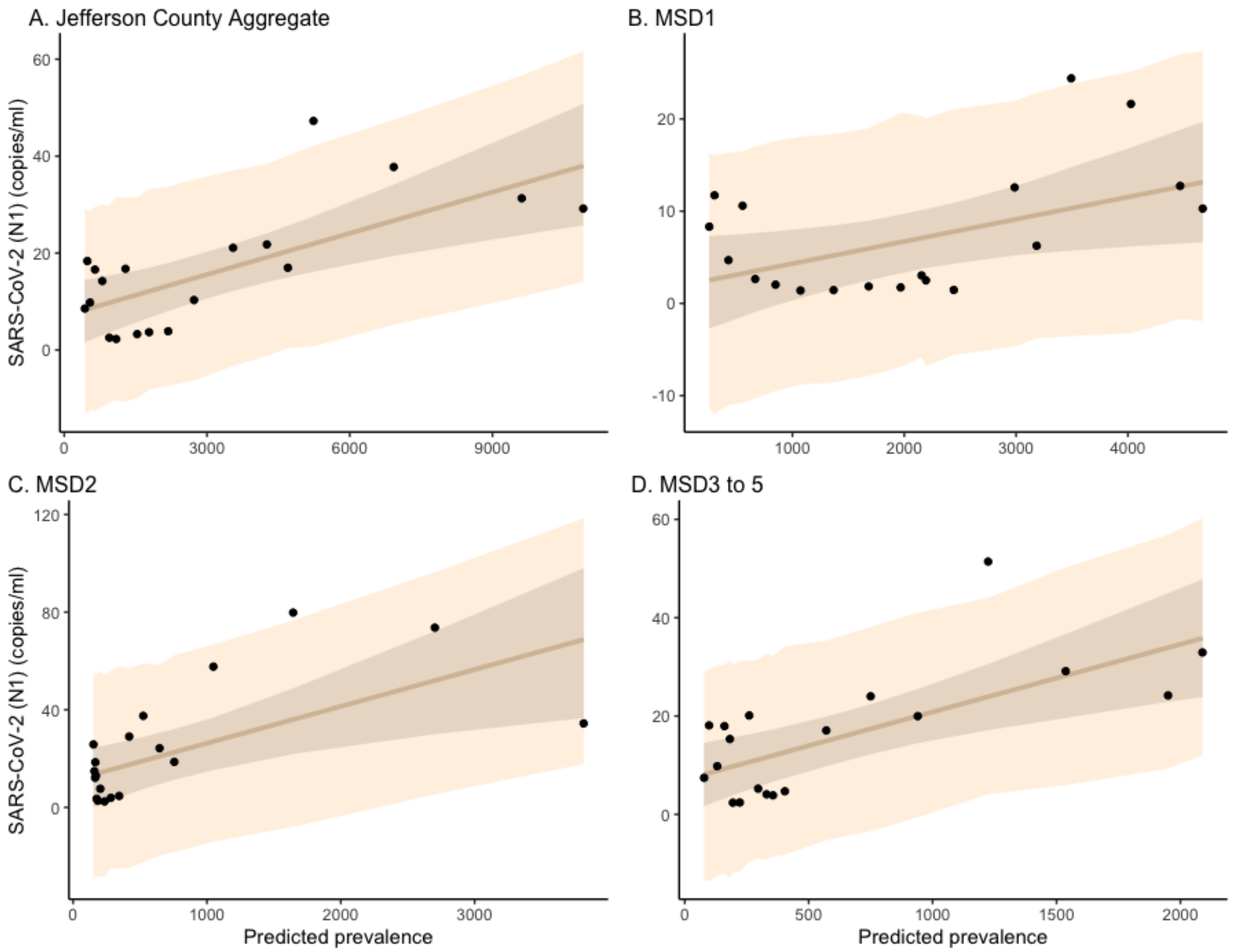

Figure S3.2. **Prevalence versus SARS-CoV-2 (N1) wastewater concentration in sewersheds of Jefferson County, KY (USA).** Bayesian regression between predicted weekly prevalence of SARS-CoV-2 infections and wastewater in the entire Jefferson County (Panel A) as well as stratified by sewershed (Panels B–D). Straight line is the fitted Bayesian regression line. The darker shade marks the 95% credible interval and lighter shade marks the 95% prediction interval.

medRxiv preprint doi: https://doi.org/10.1101/2023.01.06.23284260; this version posted January 7, 2023. The copyright holder for this preprint (which was not certified by peer review) is the author/funder, who has granted medRxiv a license to display the preprint in perpetuity.
It is made available under a CC-BY 4.0 International license .

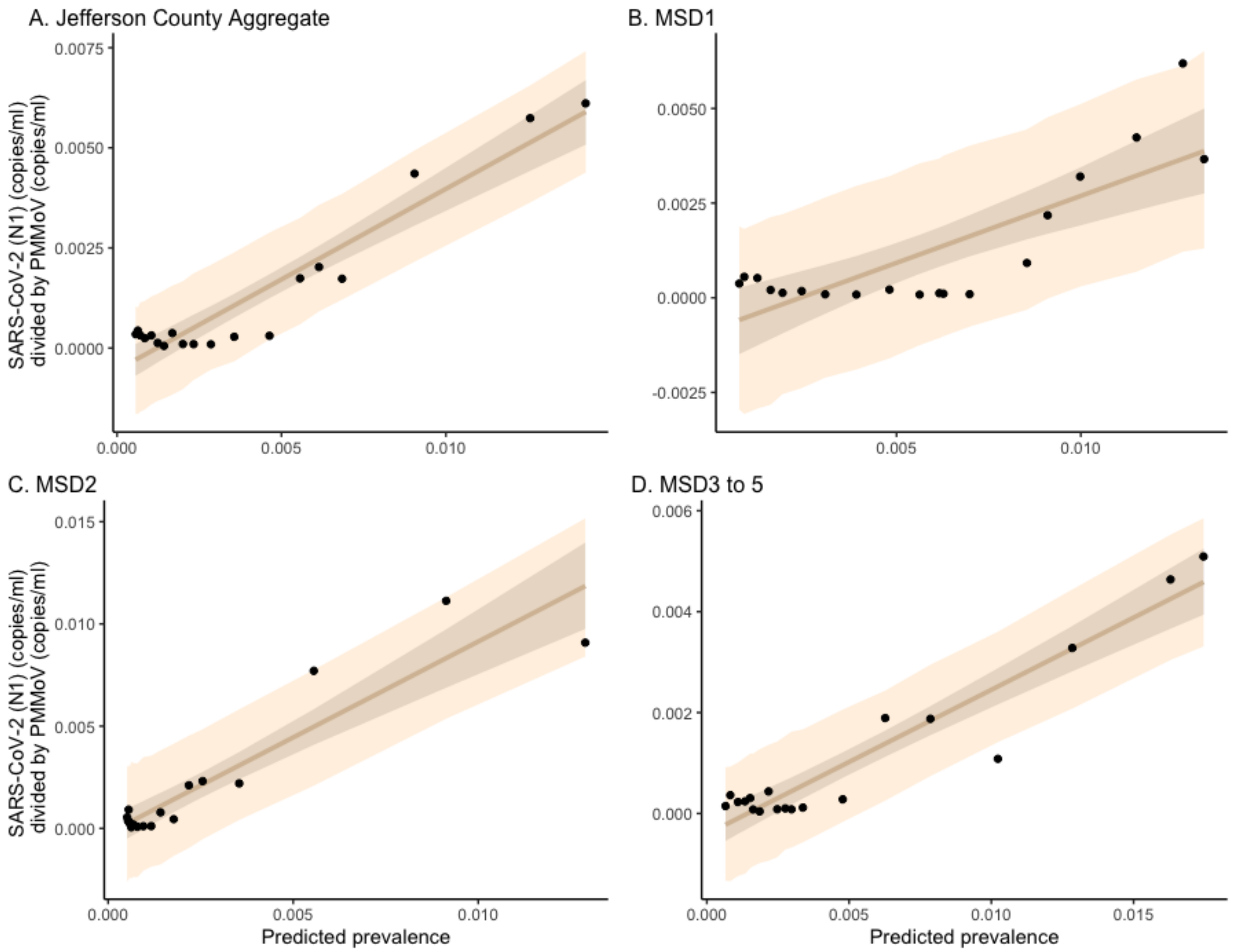

Figure S3.3. **Prevalence versus wastewater SARS-CoV-2 (N1) normalized by pepper mild mottle virus concentration in sewersheds of Jefferson County, KY (USA).** Bayesian regression between predicted weekly prevalence of SARS-CoV-2 infections and wastewater in the entire Jefferson County (Panel A) as well as stratified by sewershed (Panels B–D). Straight line is the fitted Bayesian regression line. The darker shade marks the 95% credible interval and lighter shade marks the 95% prediction interval.

medRxiv preprint doi: https://doi.org/10.1101/2023.01.06.23284260; this version posted January 7, 2023. The copyright holder for this preprint (which was not certified by peer review) is the author/funder, who has granted medRxiv a license to display the preprint in perpetuity.
It is made available under a CC-BY 4.0 International license .

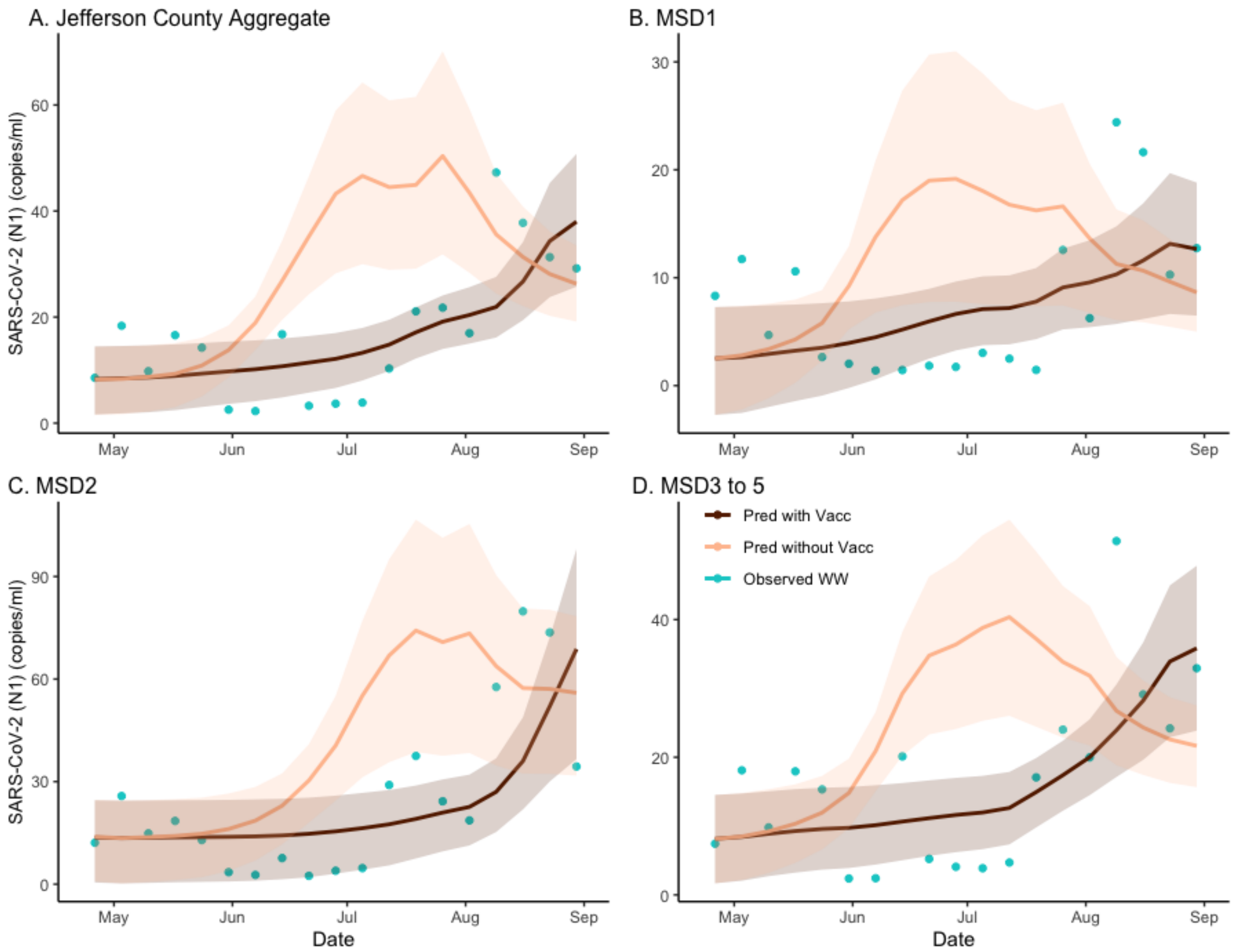

Figure S3.4. **The estimated effect of vaccination on SARS-CoV-2 wastewater concentration in sewersheds of Jefferson County, KY (USA).** The deep brown line is the regression-based fit to the wastewater concentration and the light brown line is the prediction of wastewater concentration using synthetic prevalence from the model with the Delta variant effect zeroed out. The shaded areas represent 95% credible intervals. The blue dots are observed weekly average wastewater concentration. The panels compare the mutation effect on wastewater concentration for Jefferson County (Panel A) as well as stratified by sewershed (Panels B–D).

medRxiv preprint doi: https://doi.org/10.1101/2023.01.06.23284260; this version posted January 7, 2023. The copyright holder for this preprint (which was not certified by peer review) is the author/funder, who has granted medRxiv a license to display the preprint in perpetuity. It is made available under a CC-BY 4.0 International license .

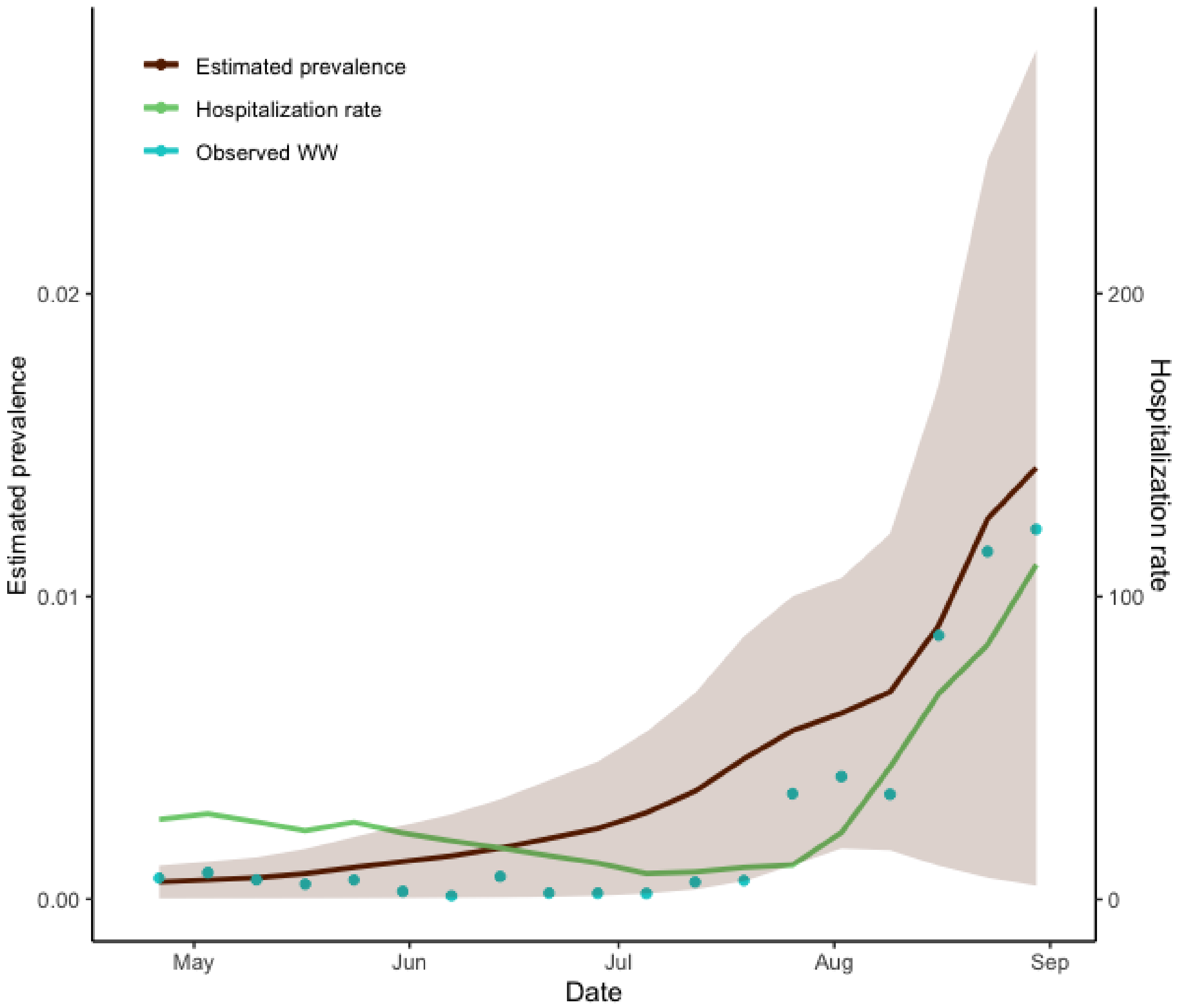

Figure S3.5. **SARS-CoV-2 prevalence and hospitalizations versus SARS-CoV-2 wastewater concentration normalized by pepper mild mottle virus, Jefferson County, KY (USA).** Relationship among observed wastewater concentration, the hospitalization rate, and estimated prevalence. The dark brown line represents the estimated prevalence, and the shaded area is the 95% credible interval of MCMC simulation. The green line is the weekly average of daily hospitalization rate of Jefferson County, and the blue dots represent the weekly average of wastewater concentrations. The Pearson correlation coefficient of estimated prevalence and wastewater concentration is 0·916 (95% CI=(0·764, 0·976)) and that of hospitalization rate and wastewater concentration is 0·720 (95% CI =(0·224, 0·953)).

medRxiv preprint doi: https://doi.org/10.1101/2023.01.06.23284260; this version posted January 7, 2023. The copyright holder for this preprint (which was not certified by peer review) is the author/funder, who has granted medRxiv a license to display the preprint in perpetuity.
It is made available under a CC-BY 4.0 International license .

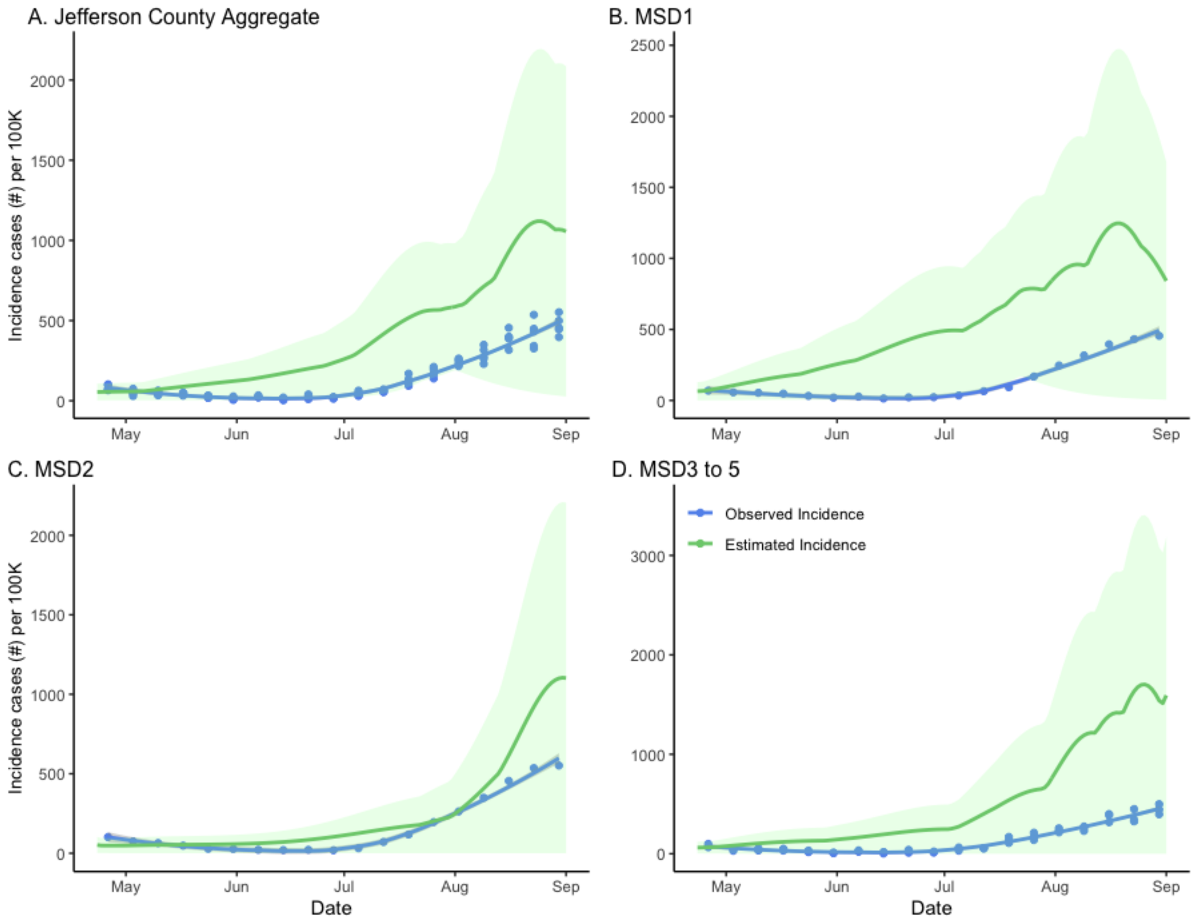

Figure S3.6. **Clinical versus estimated incidence in sewersheds of Jefferson County, KY (USA).** Posterior density and credibility bounds (green curve) of the weekly aggregated incidence rate as predicted by the model compared to official weekly incidence for Jefferson County (blue dots and trend line) as reported by the Jefferson County Health Department. The panels compare aggregated incidence for Jefferson County (Panel A) as well as stratified by sewershed (Panel B–D). The model plots are based on Hamiltonian MCMC samples, with 6000 steps and 2000 steps burn-in period.

medRxiv preprint doi: https://doi.org/10.1101/2023.01.06.23284260; this version posted January 7, 2023. The copyright holder for this preprint (which was not certified by peer review) is the author/funder, who has granted medRxiv a license to display the preprint in perpetuity.
It is made available under a CC-BY 4.0 International license .

S3.2 Details on regression model for wastewater concentration

To relate the SVI_2RT model predictions to the serial wastewater measurements of SARS-CoV-2 concentrations, the Bayesian linear regressions were performed based on aggregated county data and data stratified by sewershed area.

To obtain the linear regressions, the procedure was as follows: Let $\tilde{I}_{tj}$ be the model estimated percentage prevalence corresponding to the same week and sewershed area. W_{tj} was defined in Eq. (5). The linear and NB regression models are given by:

$$W_{tj} = \beta_0 + \beta_1 \tilde{I}_{tj} + e_{tj}, \quad (5)$$

In the Bayesian linear regression models, non-informative priors were assigned. Specifically, the non-informative Cauchy distribution was assigned to the regression coefficients, and the non-informative gamma prior was assigned to the dispersion parameter of the error term. The summary of the posterior estimates of all regression parameters is presented in Table S3.4, and fitting and prediction using the regression model are represented in Figure 2.

S3.3 Time lag-dependency between wastewater concentration and hospitalization rate

It takes a certain period for the patient to be admitted to the hospital to receive treatment. To identify the time lag-dependency between wastewater concentration and hospitalization rate, a simple linear regression analysis was performed using time-lagged variable as a predictor. Let W_{t-d} be the weekly aggregated average wastewater concentration at week t the aggregated Jefferson County, and d represents a time lag. H_t represents the hospitalization rate at time t. The regression model with time lag dependent variable is given by:

$H_t = \beta_0 + \beta_1 W_{t-d} + e_t$. (6)

In this model, we changed the time lag d from 1 to 4 so that the maximum period from a shred of evidence of the community spread of COVID-19 in wastewater to reach a burden to hospitalization is about a month. Of note, hospitalizations data is available daily while wastewater is weekly

Additionally, we performed a simulation study using this regression model how to check how much the hospitalization rate changes according to the vaccination rate. We changed the vaccination rate so that the vaccination percentage of the community was 0% and predicted the serial estimates Pred_t in Eq. (4). And then, we predicted the wastewater concentration using a linear regression model and used them as the predictor in the regression model (5).

medRxiv preprint doi: https://doi.org/10.1101/2023.01.06.23284260; this version posted January 7, 2023. The copyright holder for this preprint (which was not certified by peer review) is the author/funder, who has granted medRxiv a license to display the preprint in perpetuity.
It is made available under a CC-BY 4.0 International license .

S3.4 Calculation of effects based on factual and counter-factual scenarios

All effects of the factual and counterfactual (zero vaccinated or no Delta variant). Data are calculated using the area under the curve using factual (empirical) data and counterfactual (synthetic) data. The equation to estimate the effect is given as:

$$\left|\frac{\text{Area under counterfacutual data}}{\text{Area under factul data}} - 1\right|$$

medRxiv preprint doi: https://doi.org/10.1101/2023.01.06.23284260; this version posted January 7, 2023. The copyright holder for this preprint (which was not certified by peer review) is the author/funder, who has granted medRxiv a license to display the preprint in perpetuity.
It is made available under a CC-BY 4.0 International license .

References

1 Boyle L, Hletko S, Huang J, et al. Selective sweeps in SARS-CoV-2 variant competition. *Proc Natl Acad Sci USA* 2022; **119**(47): e2213879119. doi:10.1073/pnas.2213879119

2 Yang W, Shaman J. Development of a model-inference system for estimating epidemiological characteristics of SARS-CoV-2 variants of concern. *Nat Commun* 2021; **12**.1: 1–9. doi:10.1038/s41467-021-25913-9

3 Smith T, Holm RH, Keith RJ, et al. Quantifying the relationship between sub-population wastewater samples and community-wide SARS-CoV-2 seroprevalence. *Sci Total Environ* 2022; **853**: 158567. doi:10.1016/j.scitotenv.2022.158567

4 KhudaBukhsh WR, Choi B, Kenah E, Rempala GA. Survival dynamical systems: individual-level survival analysis from population-level epidemic models. *Interface focus* 2020; **10**(1): 20190048. doi:10.1098/rsfs.2019.0048

medRxiv preprint doi: https://doi.org/10.1101/2023.01.06.23284260; this version posted January 7, 2023. The copyright holder for this preprint (which was not certified by peer review) is the author/funder, who has granted medRxiv a license to display the preprint in perpetuity.
It is made available under a CC-BY 4.0 International license .

S4. Variant detection

Table S4.1. **Periods of Alpha and Delta variant dominance in in sewersheds of Jefferson County, KY (USA).**

Catchment site	Alpha Dominant		Delta Dominant	
	Begin Date	End Date	Begin Date	End Date
MSD01	3/30/21	5/17/21	7/12/21	8/30/21
MSD02	3/30/21	5/24/21	7/12/21	8/30/21
MSD03	3/30/21	6/21/21	7/19/21	8/30/21
MSD04	3/30/21	7/5/21	7/19/21	8/30/21
MSD05	3/30/21	6/28/21	7/26/21	8/30/21
